# VEZF1 Elements Mediate Protection from DNA Methylation

**DOI:** 10.1371/journal.pgen.1000804

**Published:** 2010-01-08

**Authors:** Jacqueline Dickson, Humaira Gowher, Ruslan Strogantsev, Miklos Gaszner, Alan Hair, Gary Felsenfeld, Adam G. West

**Affiliations:** 1 Section of Pathology and Gene Regulation, Faculty of Medicine, University of Glasgow, Western Infirmary, Glasgow, United Kingdom; 2 Laboratory of Molecular Biology, National Institute of Diabetes and Digestive and Kidney Diseases, National Institutes of Health, Bethesda, Maryland, United States of America; University of Illinois at Urbana-Champaign, United States of America

## Abstract

There is growing consensus that genome organization and long-range gene regulation involves partitioning of the genome into domains of distinct epigenetic chromatin states. Chromatin insulator or barrier elements are key components of these processes as they can establish boundaries between chromatin states. The ability of elements such as the paradigm *β-globin* HS4 insulator to block the range of enhancers or the spread of repressive histone modifications is well established. Here we have addressed the hypothesis that a barrier element in vertebrates should be capable of defending a gene from silencing by DNA methylation. Using an established stable reporter gene system, we find that HS4 acts specifically to protect a gene promoter from *de novo* DNA methylation. Notably, protection from methylation can occur in the absence of histone acetylation or transcription. There is a division of labor at HS4; the sequences that mediate protection from methylation are separable from those that mediate CTCF-dependent enhancer blocking and USF-dependent histone modification recruitment. The zinc finger protein VEZF1 was purified as the factor that specifically interacts with the methylation protection elements. VEZF1 is a candidate CpG island protection factor as the G-rich sequences bound by VEZF1 are frequently found at CpG island promoters. Indeed, we show that VEZF1 elements are sufficient to mediate demethylation and protection of the *APRT* CpG island promoter from DNA methylation. We propose that many barrier elements in vertebrates will prevent DNA methylation in addition to blocking the propagation of repressive histone modifications, as either process is sufficient to direct the establishment of an epigenetically stable silent chromatin state.

## Author Summary

DNA sequences known as chromatin insulator or barrier elements are considered key components of genome organization as they can establish boundaries between transcriptionally permissive and repressive chromatin domains. Here we address the hypothesis that barrier elements in vertebrates can protect genes from transcriptional silencing that is marked by DNA methylation. We have found that the HS4 insulator element from the *β-globin* gene locus can protect a gene promoter from DNA methylation. Protection from DNA methylation is separable from other insulator activities and is mapped to three transcription factor binding sites occupied by the zinc finger protein VEZF1, a novel chromatin barrier protein. VEZF1 is a candidate factor for the protection of promoters from DNA methylation. We found that VEZF1-specific binding sites are sufficient to mediate demethylation and protection of the *APRT* gene promoter from DNA methylation. We propose that barrier elements in vertebrates must be capable of preventing DNA methylation in addition to blocking the propagation of silencing histone modifications, as either process is sufficient to direct the establishment of an inactive chromatin state.

## Introduction

It has been proposed that genes and gene clusters are organized into chromatin domains that are maintained independent of their surroundings through the establishment of boundaries [1],[2]. These boundaries may be variable in position, resulting from a balance between countervailing chromatin opening and condensing processes. Alternatively, chromatin boundaries of fixed position could be established by specific DNA sequence elements and their associated binding proteins. Such elements, collectively called insulators, possess a common ability to protect genes from inappropriate signals emanating from their surrounding environment [3]–[7].

**Figure 1:**
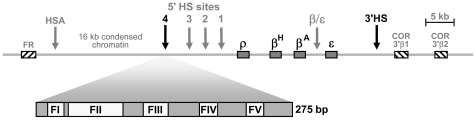
Schematic representation of the chicken *β-globin* cluster and surrounding loci. Boxes represent the folate receptor (*FR*), *β-globin* (ρ, β^H^, β^A^ and ε) and chicken olfactory receptor (*COR*) genes (not to scale). Arrows indicate DNaseI hypersensitive sites. The core of the HS4 element is expanded to show the positions of the five *in vitro* footprinted sequences [11].

The chicken *β-globin* genes are clustered within a thirty kilobase domain of nuclease accessible chromatin, the 5′ boundary of which is marked by a constitutive DNaseI hypersensitive site called HS4 (Figure 1). The HS4 element has two activities that functionally define insulators. First, it can block the action of an enhancer element on a linked promoter, but only when positioned between the two [8]. The protein CTCF mediates the enhancer blocking activity of the HS4 element [9]. Second, the HS4 insulator acts as a barrier to chromosomal position effect silencing [10]. The activities of HS4 have been mapped to a 275 bp “core” element that contains five protein binding sites revealed by DNase I footprinting [9]–[11] (Figure S1). The enhancer blocking and barrier activities of HS4 appear to have different underlying mechanisms as they are separable in assay systems. The CTCF binding site footprint II (FII) is necessary and sufficient for enhancer blocking, but can be deleted from HS4 without affecting barrier activity [9],[12],[13]. The four remaining protein binding sites are all essential for barrier activity (FI, FIII, FIV and FV) but dispensable for enhancer blocking activity [9].

We previously found that the binding of ubiquitous USF proteins to a single site in HS4, footprint FIV, is a necessary component of its barrier activity [14]. USF serves to constitutively recruit several histone modifying enzymes, leading to the enrichment of a panel of histone modifications typically associated with transcriptionally active open chromatin, including H3ac, H4ac, H3K4me2 and H4R3me2as. Knock down of USF expression abolishes the recruitment of active histone modifications and leads to the encroachment of transcriptionally repressive chromatin marked by H3K9me2 and H3K27me3 into the *β-globin* locus [14],[15]. While USF function is necessary, it is not sufficient for HS4's barrier activity. Deletion of any one of the three remaining HS4 binding sites FI, FIII or FV disrupts barrier activity without affecting USF-mediated recruitment of histone modifications to HS4 [12],[14].

We hypothesized that the FI, FIII and FV sites may contribute to barrier activity by preventing a transcriptional silencing process other than that mediated by repressive histone modifications. We previously observed that transgenes lack promoter DNA methylation when shielded from chromosomal silencing by HS4 elements [16]. Similar results were seen with retroviral transgenes shielded by HS4 [17]. It was not clear from these studies whether the lack of DNA methylation was an indirect consequence of transcriptional activity of insulated transgenes, nor did these studies address whether particular DNA elements or proteins bound at HS4 are specifically responsible for this protection. We now use insulator mutations to demonstrate that HS4 does specifically protect a gene promoter from DNA methylation. We determine which HS4 sequence elements are responsible for protection from methylation and have purified the protein that recognizes these elements *in vivo*. We also demonstrate that these elements are able to mediate the demethylation of a CpG island promoter.

## Results

### Three elements within the HS4 insulator protect a promoter from DNA methylation

To investigate whether HS4 acts specifically to counter DNA methylation, we have studied transgenic cell lines that were previously established for the HS4 barrier assay. The assay construct consists of an *IL-2R* reporter gene driven by an erythroid enhancer and promoter randomly integrated into erythroid 6C2 cells. These transgenes are susceptible to chromosomal silencing over a period of 20–40 days in culture following the removal of selection, with transgenic promoters being subject to DNA methylation and subsequent recruitment of the Mi-2/NuRD co-repressor complex [12],[16]. In contrast, transgenes flanked by wild-type HS4 insulators are protected from silencing and lack promoter DNA methylation [16],[17]. It was unclear from these earlier results whether the lack of methylation was a consequence of transcriptional activity or whether particular HS4 activities mediate protection from methylation.

**Figure 2:**
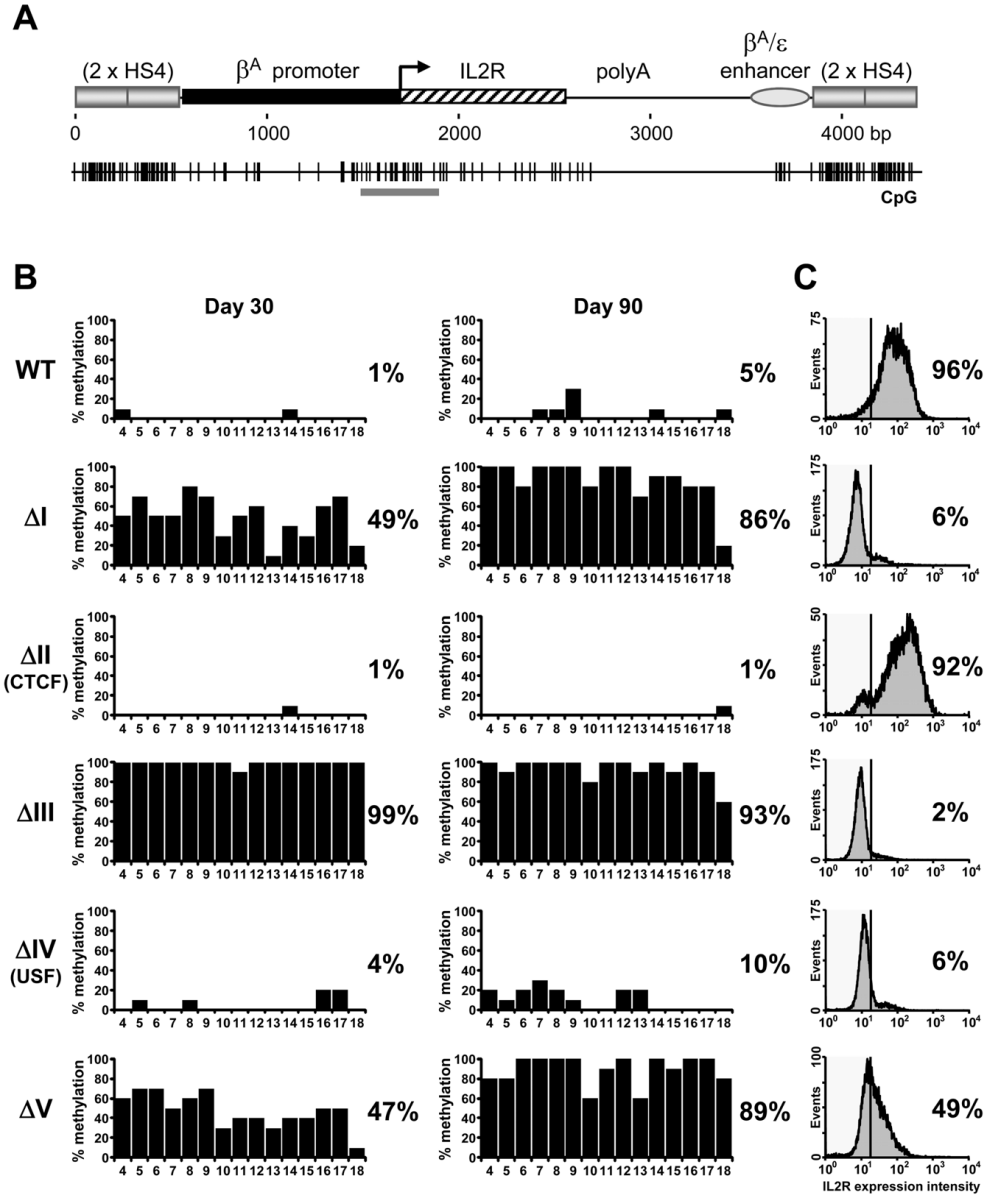
Three footprinted sites in the HS4 barrier protect a promoter from DNA methylation. (A) Schematic representation of the *IL-2R* transgene drawn to scale with the distribution of CpG dinucleotides shown below. Promoter region CpG dinucleotides subject to bisulfite genomic sequencing (BSEQ) analysis are indicated by the gray bar. (B) CpG methylation of transgene promoters flanked by wild-type or mutant HS4 insulators after 30 (left) or 90 (right) days of culture. The percentage of methylation for each CpG from 10 clones is plotted. The average methylation for all CpGs is indicated to the right of each plot. Scoring from individual clones can be found in Table S1. Numbers below each histogram refer to CpG numbering from [16], where CpG 4–11 and 12–18 reside in the promoter and coding sequence, respectively. Data are representative of two independent transgenic lines, with an average methylation variation of 7% or less. (C) Transgenic IL-2R expression for each line in (B) as monitored by flow cytometry. WT and ΔII lines retain IL-2R expression, whereas ΔI, ΔIII, ΔIV and ΔV all succumb to silencing following 20–40 days of culture.

We have performed clonal bisulfite sequencing on single/low-copy transgenes flanked by HS4 insulators that are either wild type or carry deletions in one of five protein binding site ‘footprints’ (Figure 1). We have studied the same stocks of the same cloned transgenic cells that have been characterized for long term expression and histone modification status [12],[14]. The transgene promoter remains free of DNA methylation when insulated from chromosomal silencing by wild type HS4 elements, even following prolonged culture (Figure 2B, WT). Strikingly, we find that transgenic promoters are subject to almost complete DNA methylation if any one of three footprinted sites, FI, FIII or FV, is deleted from the flanking HS4 insulators (Figure 2, Δ1, Δ3 and Δ5). These profiles of DNA hypermethylation are indistinguishable from those observed at non-insulated transgenes [16]. In contrast, deletion of the CTCF (FII) binding site from HS4 results in little *de novo* DNA methylation of the promoter (Figure 2B, ΔII). The deletion of the CTCF site has no effect on barrier activity and the lack of methylation observed is consistent with the transcriptionally active state of this transgene (Figure 2C, [12]). *De novo* DNA methylation of the transgene promoter has previously been observed to be a secondary consequence of chromosomal silencing in the absence of insulation [18]. This suggested that deletion of the USF binding site from HS4, which abolishes HS4's barrier activity and results in the loss of active histone modifications [14] and transcriptional silencing [12], should also result in promoter hypermethylation. To our surprise, deletion of the USF (FIV) binding site from HS4 results in little *de novo* DNA methylation of the promoter (Figure 2B, ΔIV). These results show that the DNA methylation status of a promoter does not necessarily follow its histone modification and transcription status. They also strongly indicate that three protein binding sites at HS4 (FI, FIII and FV) have a specific role to mediate protection from silencing associated with *de novo* DNA methylation.

### Mutant insulators are partially methylated

**Figure 3:**
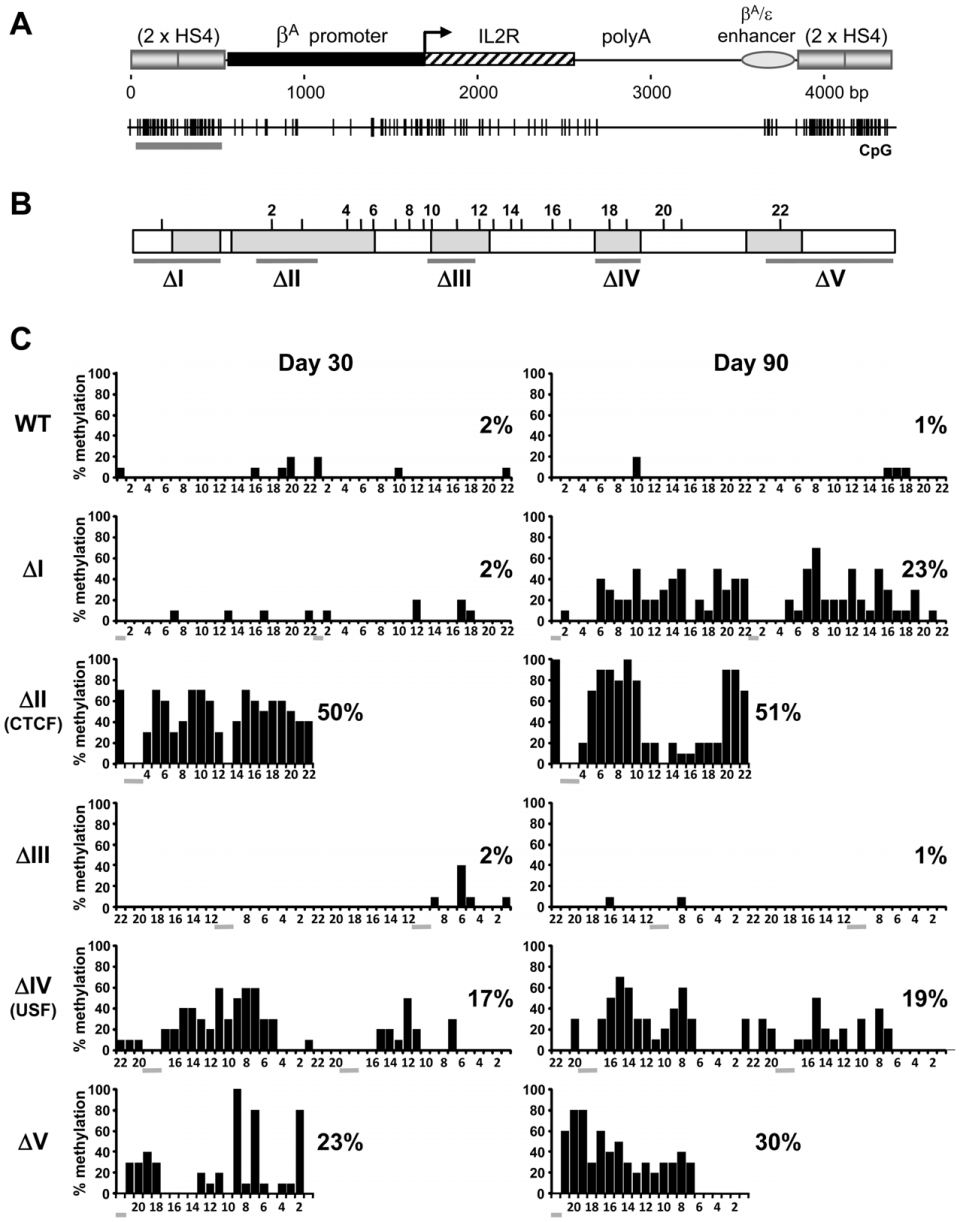
Mutations of insulator protein binding sites result in the *de novo* methylation of HS4 itself. (A) Schematic representation of the *IL-2R* transgene including the upstream HS4 elements subject to bisulfite genomic sequencing analysis (gray horizontal bar below CpG plot). (B) Schematic representation of a 275 bp HS4 core element, drawn to scale. Footprinted sequences are shaded gray. CpG locations with their assigned numbers are shown above. Horizontal bars indicate bases subject to deletion. (C) CpG methylation of wild-type (WT) or mutant (ΔI - ΔV) HS4 insulators after 30 (left) or 90 (right) days of culture. Both copies of HS4 were sequenced, except for ΔII and ΔV, where only the outermost copy was sequenced (see Materials and Methods). The percentage of methylation for each CpG from 10 clones is plotted. The average methylation for all CpGs is indicated to the right of each plot. Scoring from individual clones can be found in Table S2. Numbers below each histogram refer to CpG numbering as assigned in (B). Horizontal gray bars indicate deleted CpGs. Data are representative of at least two independent transgenic lines, with an average methylation variation of 12% or less.

We next sought to determine how HS4 footprint deletions affect the timing and level of methylation of the mutant insulators in comparison with their linked transgene promoters. The dogma established from previous studies posits that a barrier element like HS4 acts as a passive barrier to the propagation, or spreading, of chromosomal silencing. We therefore expect that when HS4 mutations compromise barrier activity, the mutant insualtors would become methylated either prior to, or coincident with, the transgene promoters they are shielding. We performed bisulfite sequencing of the HS4 elements that are located 5′ of the *IL-2R* transgenes (Figure 3A). Analyses were made following 30 days of culture, typically the period at which epigenetic silencing of the transgene is being established in non-insulated lines, and after 90 days, at which point any silencing will be complete. We find that wild type HS4 elements remain unmethylated during long term culture, concordant with the lack of transgene methylation (Figure 3C, WT). Deletion of the FI and FV sites results in partial methylation of HS4 (Figure 3C, ΔI and ΔV). This is in line with the effects of these mutations on promoter methylation. The timing of methylation does not fit the spreading models however, as the transgene promoter becomes methylated prior to the flanking insulator (compare ΔI at day 30 in Figure 2B with that in Figure 3C). Furthermore, deletion of the FIII site does not result in methylation of HS4 despite complete promoter methylation resulting from this mutation (compare ΔIII at day 90 in Figure 3C with that in Figure 2B). We also found that deletion of either the CTCF or USF sites leads to partial methylation of HS4, but with no methylation at the promoter (compare ΔII and ΔIV at day 90 in Figure 3C with that in Figure 2B).

We note that the patterns of partial methylation of mutant HS4 elements are heterogeneous, with none of the individually sequenced clones becoming densely methylated (Table S2). The partial methylation of mutant HS4 elements (20%–50%) contrasts with the near total DNA methylation observed at the silenced promoters flanked by FI, FIII or FV site mutant insulators (90%–100%). These findings reveal a disconnect between the level and timing of *de novo* DNA methylation at a transgene and flanking insulators.

### Identification of HS4-binding activities

**Figure 4:**
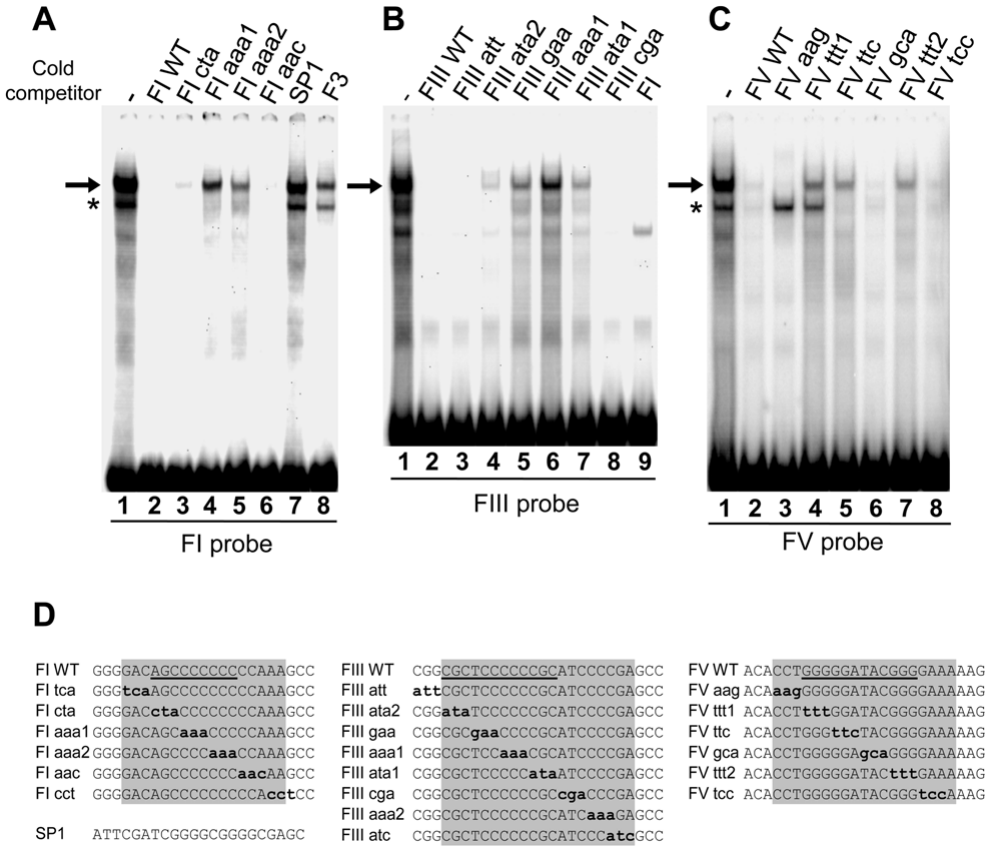
Nuclear proteins specifically interact with the dG-dC strings at HS4 footprints I, III, and V. (A–C) Gel mobility shift analysis of interactions between chicken adult erythrocyte nuclear extract and ^32^P-labelled FI (A), FIII (B), and FV (C) oligonucleotide duplexes. Unlabelled competitor duplexes (indicated above each lane) were added at 50 fold molar excess. Arrows indicate footprint sequence-specific complexes. Asterisks indicate a non-specific FI complex in (A) and an FV complex that was not pursued further due to inconsistent abundance in (C). (D) Sequences present in competitor oligonucleotides. Mutations shown in bold lower case. Footprinted bases are indicated by shading. Bases that are essential for maximal binding are underlined; mutations of other bases had no effect on binding (A–C, data not shown).

The footprinted sequences FI, FIII and FV are specifically required for HS4's ability to protect against DNA-methylation-mediated silencing of a transgene. We wished to identify the factors that interact with each of the FI, FIII and FV sites to better understand this activity. We established gel mobility shift assays for insulator-binding activities using nuclear protein extracts of the chicken early erythroid cell line 6C2 (the cell line in which the barrier assay is performed) and adult chicken red blood cells (an abundant source of nuclear protein for purification purposes). Complexes of similar mobility and intensity are observed between the two nuclear extracts and each of the FI, FIII and FV sites (Figure 4, 6C2 data not shown). Competition assays show that these complexes are all specific for homopolymeric dG-dC strings found in each site. Unlabelled wild type FI duplexes compete efficiently with the formation of the major complex with FI, whereas FI duplexes harboring mutations in the (dG-dC)_9_ string are much less effective as competitors (Figure 4A, arrow, compare lanes 2, 4 and 5). The major complex with FIII specifically interacts with the (dG-dC)_6_ string at its center (Figure 4B, compare lanes 1, 5, 6 and 7) and the major FV complex also specifically interacts with bases in both of its (dG-dC) strings (Figure 4C, compare lanes 1, 4, 5 and 7).

The same proteins interact with the FI, FIII and FV sites. This is supported by the observation that the three sites can efficiently compete with each other. Unlabelled FIII duplexes compete for nuclear protein interactions with labeled FI (Figure 4A, compare lanes 1 and 8) and FI duplexes efficiently compete for interaction with FIII (Figure 4B, compare lanes 1 and 9), for example. Mutational analysis shows that this cross-competition is dependent upon the dG-dC string bases within each footprint site (data not shown). Competition assays also reveal that the relative affinity of nuclear proteins for the three sites differs somewhat. FI complexes form with approximately 2- and 5-fold greater affinity than FIII and FV complexes, respectively (data not shown). Together, these observations of similar sequence specificity and comparable complex mobilities indicate that common nuclear proteins interact with all of these sites.

### VEZF1 specifically interacts with G-rich sites in the HS4 insulator and the *β^A^-globin* promoter

**Figure 5:**
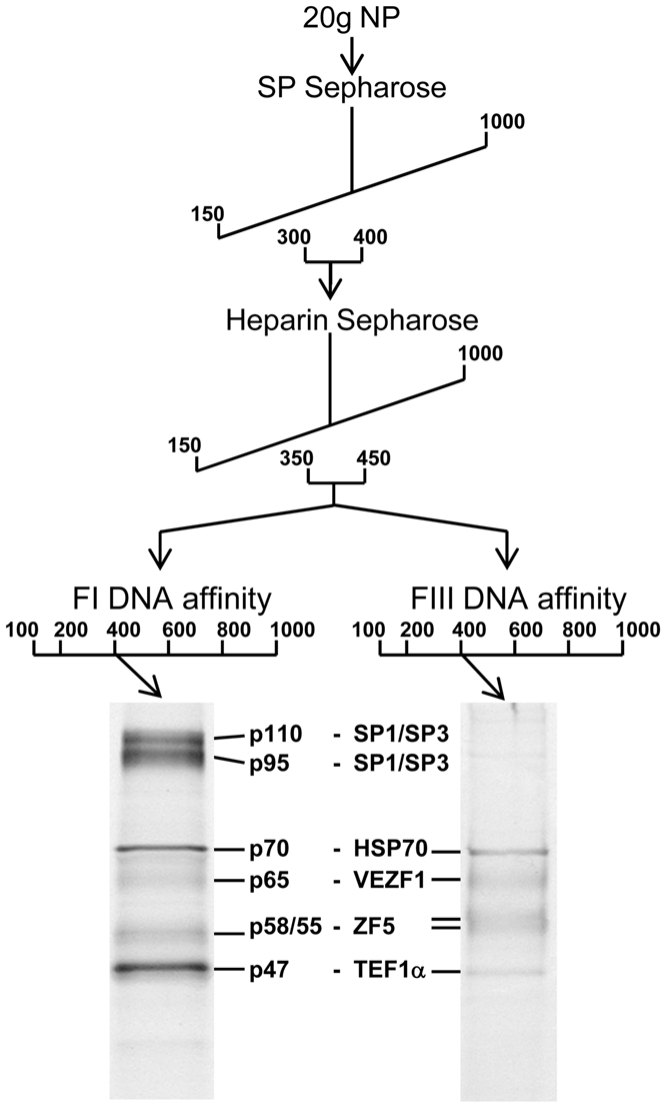
Purification of FI- and FIII-binding proteins. Schematic representation of the steps used to purify DNA-binding proteins from adult erythrocyte nuclear protein (NP) extracts that interact with FI and FIII sequences. Numbers in the workflow indicate the salt concentration (mM) at which DNA binding factors eluted from each column. DNA binding activity was tracked using gel mobility shift analysis and the sequence specificity of complex-forming factors was checked by competition analysis following each purification stage (data not shown). FI- and FIII-binding activities exactly co-purified. The eluate pool from the Heparin step was split in two prior to either FI or FIII DNA affinity chromatography. SDS-PAGE of proteins eluted from DNA affinity columns at 400 mM KCl visualized with colloidal Coomassie is shown below. The size of each polypeptide (kDa) is indicated.

We purified proteins that specifically interact with FI and FIII from chicken red blood cells by conventional chromatography. FI- and FIII-binding activities exactly co-fractionated following ion exchange chromatography with SP- and Heparin sepharose (Figure 5). The active elution peak from Heparin sepharose chromatography was split in two and fractionated in parallel by either FI or FIII DNA affinity chromatography. The resulting purified polypeptides were sequenced by tandem mass spectrometry (Figure S2). The proteins Hsp70, VEZF1, ZF5 and TEF1α were present in both FI and FIII DNA affinity eluates. The proteins SP1 and SP3 were additionally present in the FI DNA affinity eluate.

**Figure 6:**
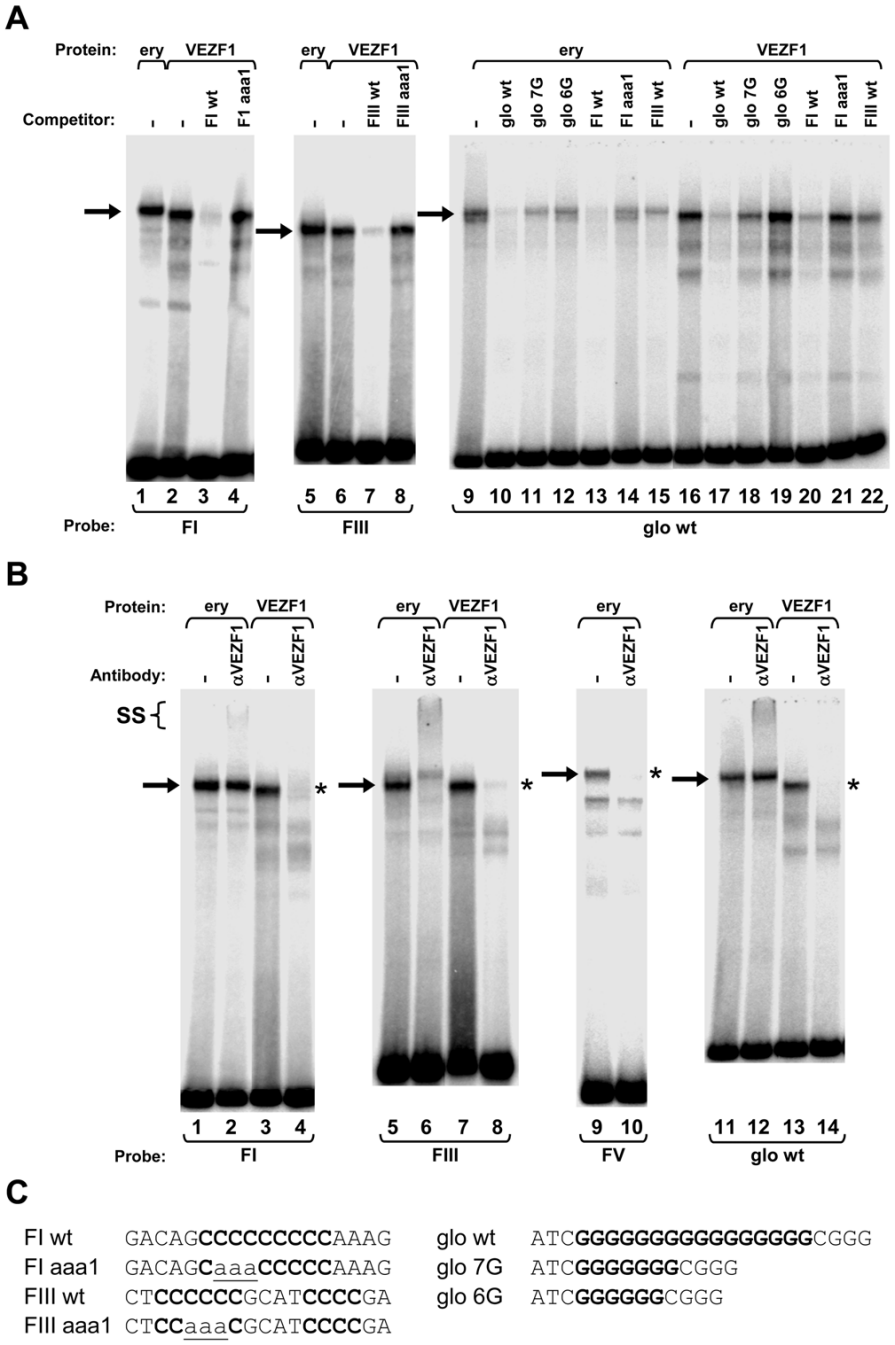
VEZF1 specifically interacts with dG–dC strings within HS4 footprints I and III and the *β^A^-globin* promoter. (A) Gel mobility shift analysis of interactions with ^32^P-labelled HS4 FI, and FIII, or *β^A^-globin* promoter ‘glo wt’ oligonucleotide duplexes. Unlabelled competitor duplexes (indicated above each lane) were added at 50 fold molar excess. Adult chicken erythrocyte nuclear extract (ery) or recombinant chicken VEZF1 were used as indicated by brackets above the lanes. Arrows indicate footprint sequence-specific complexes. (B) Gel mobility supershift assays using ^32^P-labelled FI, FIII, FV, and glo wt oligonucleotide duplexes. Proteins were pre-incubated with anti-VEZF1 antibodies (indicated above each lane) prior to incubation with DNA. VEZF1 supershifts are evidenced by either abrogation of specific complexes (asterisks) and/or formation of low mobility ternary complexes (SS). Antibodies alone do not give rise to complexes with any of the duplexes used (data not shown). Supershift experiments shown are cropped from the full gels shown in Figure S3. (C) Sequences present in oligonucleotides used in gel mobility shift assays. dG–dC strings and their mutations are bold and underlined, respectively.

We firstly cloned chicken VEZF1 (Refseq NM_001037827.1) and determined whether it interacts with the G-rich footprinted sites of the HS4 insulator, as it was previously reported that human VEZF1 (also known as DB1) interacts with similar G-rich sites [19],[20]. We find that *in vitro* translation of chicken VEZF1 cDNA yields a 65 kDa protein that efficiently interacts with the FI, FIII and FV sites (Figure 6A). The complexes formed with recombinant VEZF1 migrate slightly faster than those formed with nuclear extract. This may be a reflection of differing post translational modifications. Nonetheless, recombinant VEZF1 binds with an identical specificity to that observed for nuclear extract proteins. For example, competition of VEZF1 complexes with unlabelled FI and FIII duplexes is disrupted by mutations within their dG-dC strings (Figure 6A, lanes 1–8).

The (dG-dC) strings present in the VEZF1 sites at HS4 are reminiscent of a site in the chicken *β^A^-globin* promoter that contains a (dG-dC)_16_ string. A nuclear factor called Beta Globin Protein 1 (BGP1) was previously characterized as interacting with this site [21]. This BGP1 binding site has no effect on transcription in transient assays or on chromatinized templates *in vitro*, but was considered to indirectly assist in activation by directing nucleosome placement [22]–[24]. BGP1 protein of 66 kDa can be purified using poly(dG)-poly(dC) affinity chromatography [25]. We have now sequenced a purified BGP1 sample (a gift from J. Allan, University of Edinburgh) and find it to be VEZF1. We find that recombinant chicken VEZF1 interacts with the β^A^ promoter site with an identical specificity to that of erythrocyte nuclear protein(s) (Figure 6A, compare lanes 9–15 with 16–22). At least seven contiguous homopolymeric dG-dC base pairs are required for the efficient formation of complexes between recombinant VEZF1 or nuclear proteins and the β^A^ promoter site [25]. Consistent with this, interaction between VEZF1 and the β^A^ site is competed by unlabelled FI which contains a (dG-dC)_9_ string (Figure 6A, compare lanes 16 and 20). This competition is disrupted by mutation at the centre of the FI dG-dC string (Figure 6A, compare lanes 20 and 21). However, VEZF1 interaction with the β^A^ site is also competed by FIII which contains only a (dG-dC)_6_ string (Figure 6A, compare lanes 16 and 22). FIII contains a second short (dG-dC)_4_ string, as does FV (see figure 4D), which may compensate to form a bipartite recognition motif. Recombinant VEZF1 interacts with the contiguous dG-dC strings of the FI and β^A^ sites with approximately 2- and 5-fold greater affinities than the bipartite dG-dC strings of the FIII and FV sites, respectively (data not shown).

Polyclonal antibodies were raised against a conserved C-terminal fragment of VEZF1, which specifically recognize the 65 kDa VEZF1 polypeptide from chicken nuclear extracts (Figure S7B). VEZF1 antibodies readily supershift/abrogate complexes between recombinant VEZF1 and the FI, FIII, FV and β^A^ sites (Figure 6B). Supershift analysis also reveals that VEZF1 is present in complexes between nuclear extracts and the FI, FIII, FV and β^A^ sites (Figure 6B). VEZF1 appears to be the only factor that interacts with the FIII and FV sites, whereas other factors also appear to bind to the FI and β^A^ sites *in vitro*.

**Figure 7:**
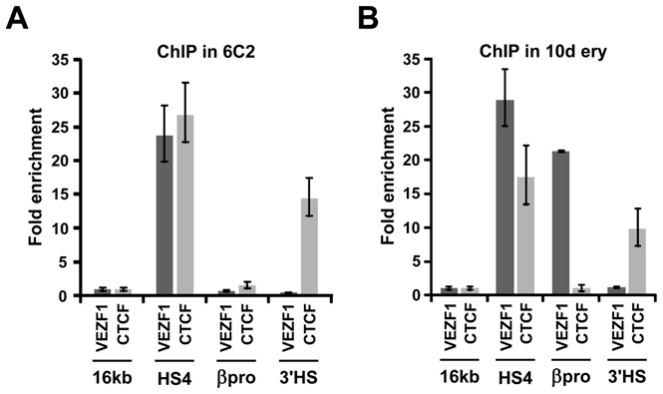
VEZF1 interacts with the HS4 insulator *in vivo*. (A) ChIP analysis of VEZF1 and CTCF interactions at the *β-globin* locus in (A) 6C2 erythroid progenitor cells or (B) 10 day embryonic erythrocytes. DNA enrichments at the β^A^ promoter or the HS4 and 3′HS insulators were normalized to a negative control located in the 16 kb condensed chromatin region upstream of the *β-globin* locus.

Chromatin immunoprecipitation (ChIP) analyses were performed to analyze the binding of VEZF1 at the chicken *β-globin* locus *in vivo*. Chromatin was prepared from the early erythroid line 6C2, which does not express β-globin and nucleated erythrocytes isolated from 10 day chicken embryos, a stage at which approximately 80% of erythrocytes are definitive and express the *β^A^-globin* gene. VEZF1 was found to strongly interact with HS4 in both 6C2 cells and erythrocytes, consistent with our *in vitro* analyses (Figure 7A and 7B). VEZF1 binding to HS4 therefore does not coincide with transcription of the *β-globin* genes. VEZF1 does not interact with the 3′HS enhancer blocking element, which does not contain any dG-dC string like motifs and lacks barrier activity [12]. This is in contrast to CTCF, which interacts strongly with both the HS4 and 3′HS insulators in 6C2 cells and erythrocytes (Figure 7A and 7B). We also find that VEZF1 strongly interacts with the β^A^ promoter, consistent with gel mobility shift assays. In contrast to HS4, VEZF1 binding to the β^A^ promoter appears to be restricted to erythrocytes in which the β^A^ gene is expressed (Figure 7A and 7B). None of the other candidate HS4-binding proteins isolated by DNA affinity purification were found to bind *in vivo* (described in Text S1) (see also Figures S3, S4, S5).

We tested whether VEZF1 requires all three of its sites for binding to HS4 *in vivo*, as all three VEZF1 binding sites are required for protection from DNA methylation. We found that this was not the case, as VEZF1 remains tightly bound at HS4 when any one of its binding sites is deleted (Figure S6). VEZF1 also remains bound to mutant HS4 elements that have become partially methylated. Consistent with this, we found that VEZF1 binding to its three sites at HS4 is not affected by CpG methylation *in vitro* (data not shown). We have attempted to disrupt VEZF1 function at HS4 following knockdown by RNAi. We strived to achieve substantial knockdown of VEZF1 to disrupt its binding to the high affinity sites at HS4. Prolonged knockdown was also required as we have previously found that the *de novo* DNA methylation of these transgenes is a gradual process that takes many days to establish [18]. We were able to knockdown VEZF1 protein to 3% of wild type levels following 2 weeks of stable miRNA expression. However, ChIP analysis revealed that VEZF1 binding to HS4 was not significantly affected following this prolonged and substantial knockdown (Figure S7). Consequently, we observed no *de novo* DNA methylation of the HS4 element and no change in HS4's ability to protect a transgene from silencing during this period (data not shown). The inadequacy of RNAi to strip constitutive transcription factor binding from high affinity sites has also been observed for CTCF [26]. Unfortunately, we are unable to study the role of murine Vezf1's role in protection from DNA methylation in *Vezf1* null ES cells, as we recently found that they are defective for *de novo* DNA methylation due to the requirement of *Vezf1* for full transcriptional activity of the *Dnmt3b* gene in these cells [27].

### VEZF1 elements protect a CpG island from *de novo* DNA methylation

**Figure 8:**
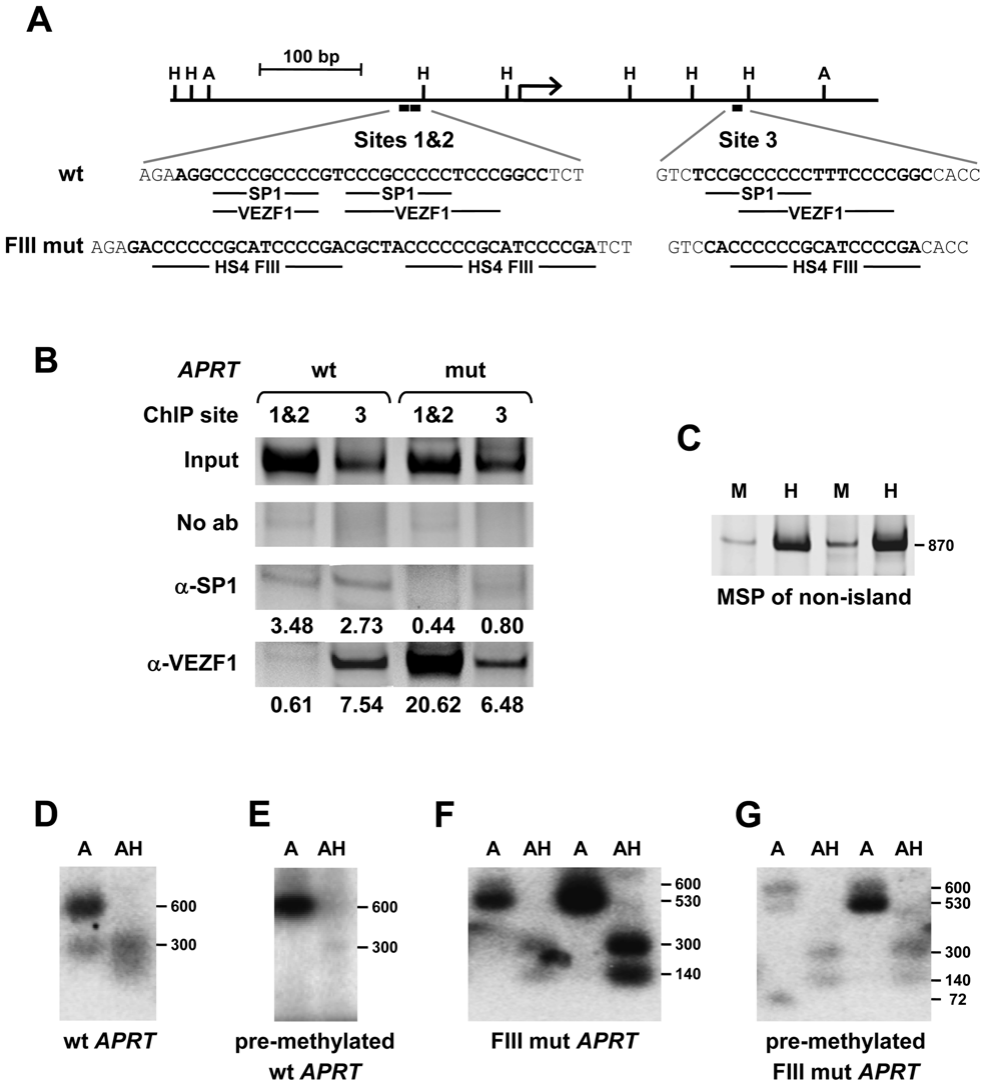
VEZF1 elements protect a CpG island from *de novo* DNA methylation. (A) Scale representation of the 700 bp hamster *APRT* CpG island (CGI). The positions of *Ava*II (A) and *Hpa*II (H) restriction sites are indicated. The locations of putative SP1 and VEZF1 binding motifs in the wild type (wt) sequence are shown. The sequence alterations in the mutant (mut) are shown beneath, where all three SP1/VEZF1 motifs are replaced with the VEZF1-specific footprint III (CCCCCCGCATCCCCGA) sequence from the HS4 insulator. (B) ChIP analysis of SP1 and VEZF1 interactions with stably integrated hamster *APRT* CGI sequences in mouse ES cells. PCR primers flanking either sites 1 and 2 (1&2) or site 3 were used to detect interactions with either the wild type (wt) *APRT* CGI or the mutant (FIII mut) containing VEZF1-specific sites. PCR products shown are ∼180 bp in size. DNA enrichments relative to input and normalized to no antibody control are shown (C) Methylation specific PCR (MSP) analysis of a stably integrated non-island region of the *APRT* locus. Genomic DNA was digested with either of the isoschizomers *Msp*I (M) or CpG methylation-sensitive *Hpa*II (H), followed by PCR over the CpG-containing sites. Increased PCR product from *Hpa*II relative to *Msp*I digested DNA demonstrates the presence of *de novo* methylation at these sites in ES cells. Two representative lines are shown. (D–G) VEZF1 sites are sufficient for the demethylation of the *APRT* CpG island. Southern blotting of *APRT* CpG island elements stably integrated into ES cells. Genomic DNA was digested with *Ava*II alone (A) or with *Ava*II and *Hpa*II (AH). Digestion of the ∼600 bp *Ava*II parent fragments with *Hpa*II indicates the absence of DNA methylation. (D,E) Representative lines harboring the wild type *APRT* CGI that was unmethylated or *in vitro* methylated with M.*Sss*I prior to integration. (F,G) Two representative lines harboring the mutant *APRT* CGI, whose SP1 motifs are substituted with VEZF1-specific FIII sites from HS4, that were unmethylated or pre-methylated prior to integration.

To address whether VEZF1 elements also protect CpG island (CGI) promoters from DNA methylation, we investigated VEZF1 binding to the *APRT* gene promoter and its effects on methylation. SP1-like binding elements have been shown to be required to prevent methylation of the mouse and hamster *APRT* CpG island elements: two earlier papers have shown that deletion of the SP1-like elements is sufficient to induce methylation in these islands [28],[29]. Furthermore, pre-methylated fragments of the hamster *APRT* CGI that contain three SP1-like elements are subject to demethylation upon integration into mouse ES cells [28]. The SP1 transcription factor itself is not required for the unmethylated state of CGIs however, with the *APRT* gene remaining unmethylated and expressed normally in *Sp1* null ES cells and embryos [30]. Given that VEZF1 recognizes G-rich sequences that are similar to SP1 motifs, we hypothesized that VEZF1 may interact with the *APRT* CGI elements (Figure 8A). We performed ChIP analyses for the binding of SP1 and VEZF1 to the 720 bp hamster *APRT* CGI stably integrated into mouse ES cells (Figure 8B). We found SP1 binding at both sites 1/2 and site 3, but site 3 was also occupied by VEZF1. Supershift analysis also shows VEZF1 interaction with site 3 *in vitro* (Figure S8). Site 3 contains a motif (CCCCCCTTTCCCC) that is reminiscent of the VEZF1-specific bipartite footprint III site found at the HS4 insulator element (CCCCCCGCATCCCC).

To address whether VEZF1 elements could protect the *APRT* CGI from methylation, we replaced each of the three SP1-like elements with the VEZF1-specific FIII element from the HS4 insulator (Figure 8A). ChIP analysis shows that VEZF1 binding replaces that of SP1 at the mutant *APRT* CGI integrated into ES cells (Figure 8B). Supershift analysis also shows that VEZF1 interacts with the mutant sites 1&2 and site 3, while SP1/SP3 binding is lost (Figure S8). We then tested the ability of wild type and mutant *APRT* CGIs to resist DNA methylation. Firstly, we confirmed that the *de novo* DNA methylation machinery functioned normally in the ES cells as non-island sequences from the *APRT* gene body succumb to *de novo* methylation (Figure 8C). Consistent with previous results [28],[29], we find that the wild type *APRT* CGI is protected from DNA methylation when stably integrated into ES cells (Figure 8D). Furthermore, a pre-methylated wild type *APRT* CGI is demethylated upon integration (Figure 8E). It has previously been shown that mutation of the SP1-like elements results in the *de novo* methylation of the *APRT* CGI [28]. Our results show that substitution of the SP1-like elements with VEZF1-specific FIII elements from HS4 restores the ability of the mutant *APRT* CGI to be both protected from *de novo* methylation and to remove pre-methylation (Figure 8E and 8F). VEZF1 elements from HS4 are therefore sufficient to mediate the demethylation and protection of a CGI from DNA methylation.

## Discussion

Here we have studied the paradigm HS4 element to address our hypothesis that a barrier element in vertebrates must be capable of defending a gene from silencing by DNA methylation and have identified a novel CpG island factor. We have presented six findings: 1) a vertebrate barrier element protects a gene promoter from DNA methylation-mediated silencing, 2) the essential transcription factor VEZF1 is a barrier/anti-methylation factor, 3) there is a modular division of labor at the compound HS4 insulator as VEZF1-mediated protection from methylation is separable from CTCF-mediated enhancer blocking and USF-mediated recruitment of active histone modifications, 4) the *de novo* DNA methylation activity prevented by the HS4 barrier does not appear to spread from its chromosomal neighborhood, 5) a promoter can be protected from DNA methylation even when it lacks active histone modifications and transcriptional activity, and 6) short DNA elements bound by VEZF1 mediate the demethylation and protection of a CpG island from DNA methylation.

### The barrier activity of HS4 consists of separable activities which prevent silencing mediated by either histone or DNA methylation

**Figure 9:**
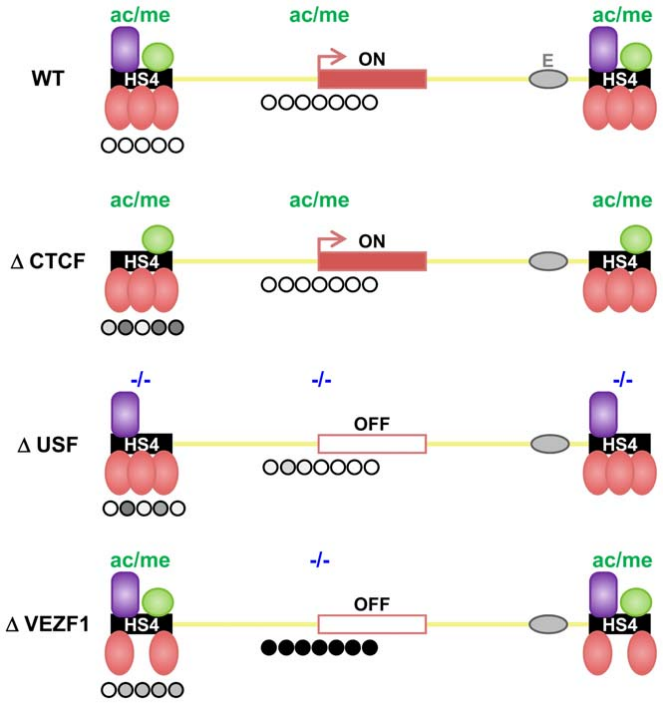
Shielding of a transgene by the multi-component HS4 barrier element. The observed effects of insulator protein binding site mutations on the DNA methylation (this study), histone modification [14] and transcriptional status (this study and [12]) of a transgene insulated by HS4. A schematic representation is shown where HS4 harbors one CTCF (purple), one USF (green) and three VEZF1 (red) binding sites. Transcriptional activity of the reporter gene is indicated with an arrow. The histone modification status is depicted above each transgene, where high and minimal levels of H3ac and H3K4me2 are represented as ac/me and -/-, respectively. The DNA methylation status of the promoter and upstream insulators is indicated below each transgene where open and filled circles represent unmethylated and methylated bases, respectively. Shades of methylation are an approximation of the data presented in Figure 1 and Figure 2. Mutation of the CTCF site disrupts enhancer blocking activity [9] but has no effect on barrier activity of HS4. USF site mutation disrupts the recruitment of active histone modifications, resulting in transcriptional silencing. The promoter remains unmethylated in USF mutants due to the action of VEZF1. VEZF1 site mutations abrogate barrier activity despite USF-mediated recruitment of histone modifications remaining intact. VEZF1 mutants are characterized by complete promoter methylation.

We have previously demonstrated that the HS4 insulator acts as a barrier to the spread of histone methylation marks associated with repressive chromatin [14],[15]. While we found that active histone modifications recruited by USF proteins are an essential component of HS4's barrier activity, they are not sufficient [14]. Three addition protein binding sites are essential for barrier activity but are not required for the recruitment of active histone modifications [12],[14]. These findings, summarized in Figure 9, indicated that there was an additional and separable component to HS4's barrier activity. Here, we show that all three sites are bound by VEZF1 and are required for HS4's ability to protect a linked promoter from *de novo* DNA methylation.

It was previously shown that the transgenes used in this study become marked by dense promoter DNA methylation upon chromosomal position effect silencing [16]. Promoter DNA methylation occurred subsequent to histone deacetylation and transcriptional inactivation of the promoter [18]. While flanking HS4 elements perfectly shield the transgene from silencing and DNA methylation, it was unclear from these experiments whether the lack of promoter methylation was simply a readout of the promoter's transcription status. We show that VEZF1-mediated protection from DNA methylation of a transgene promoter is retained even when USF site mutations at HS4 lead to histone deacetylation and transcriptional silencing (Figure 9, ΔUSF). The separation of DNA methylation protection from a promoter's histone modification and transcriptional status is a strong indication that the VEZF1 sites at HS4 possess a *bona fide* activity that is protective against DNA methylation.

### *De novo* DNA methylation of a transgene does not appear to propagate via a continuous DNA methylation-dependent spreading mechanism

Determining the source of *de novo* DNA methylation is key to our understanding of how VEZF1 binding at HS4 could protect a promoter from epigenetic silencing. Previous studies using the same transgene system studied here found that non-insulated transgenes, regardless of integration site, are consistently subject to promoter methylation upon chromosomal silencing, and that flanking with HS4 elements can shield transgenes from this methylation [16],[18]. The simplest explanation of these results is that HS4 is acting as a barrier to the encroachment, or spreading, of a silencing mechanisms that results in DNA methylation. The ability of repressive histone modifications and associated chromatin factors to mediate the spreading of gene silencing is well documented for many systems [31]. In the case of the chicken *β-globin* locus, the spreading of repressive histone modifications is observed upon perturbation of active histone modification recruitment at the HS4 barrier [14],[15]. Analyses of progressive CpG island methylation during tumor progression are consistent with models that describe the spreading of DNA methylation [32],[33].

Should *de novo* DNA methylation arise via spreading from the chromosomal integration site in our transgene system, we would expect to see high levels of methylation at compromised mutant insulators either coincident with, or prior to promoter methylation. However, we observe that promoters become methylated prior to the insulators, which remain unmethylated or become partially methylated. The observed independence of methylation states between insulator and promoter argue against spreading and clearly show that there can be no single mechanism that controls the methylation state of both the insulator and promoter. It remains possible that VEZF1 elements at HS4 are acting as a barrier to the spreading of a DNA methylation mechanism, but that additional processes prevent the accumulation of methylation at HS4 itself. An alternative possibility is that DNA methylation does not result from spreading, and that the insulator directly interacts with the promoter to deliver VEZF1 co-factors that prevent promoter methylation. In this model, the promoter itself would have its own program to recruit *de novo* DNA methylation, and VEZF1 would act as a factor that mediates inhibition of methylation. This would distinguish the activity of VEZF1 from those of USF1/USF2, which bind elsewhere in the insulator element and recruit a number of enzymes that deliver active histone modifications to the reporter gene [14],[15],[34]. It will be of interest in future to determine whether VEZF1 elements potentiate the expression of nearby genes through the control of DNA methylation.

### Intact insulator protein complexes maintain HS4 as a CpG island

The 275 bp “core” HS4 element comprises a CpG island (CGI) that is free of DNA methylation regardless of neighboring gene expression [11],[35],[36], as well as when it is inserted into the mouse *Igf2/H19* domain [37]. Consistent with this, we found that wild type transgenic HS4 elements remain unmethylated during long term culture. The processes that maintain the unmethylated state of the insulator appear to be complex, as we find that the mutation of all insulator protein binding sites results in some degree of *de novo* DNA methylation of HS4. It has previously been shown that DNA binding proteins can prevent the methylation of their binding sites simply by steric hindrance of *de novo* DNA methyltransferases (DNMTs) [38]. It is possible that the HS4 deletions studied here disrupt cooperative interactions between insulator proteins, thus permitting DNMT access. We performed ChIP experiments on HS4 mutants and found that deletion of any one insulator binding site does not lead to the loss of binding of another (Figure S6). This is in agreement with functional assays which also found that deletion of any one insulator protein binding site does not lead to the loss of function associated with another site [9],[12],[14]. These findings argue against a simple steric protection of HS4 DNA from DNMTs by transcription factor binding. The degree of methylation at mutant HS4 elements was typically moderate (20–50%) and did not increase to the near total methylation seen at the transgene promoter (90–100%) following long term culture. These observations are consistent with a balance between activities that add and remove DNA methylation at HS4.

### VEZF1 elements at CpG island promoters

All constitutively expressed genes and ∼40% of genes with tissue-restricted expression have CGI promoters [39]. CGIs are typically unmethylated, especially in the germ line, which ensures that these CpGs are not subject to mutation by spontaneous deamination. It remains to be determined how CGIs resist global *de novo* methylation during early development, and how they remain hypomethylated irrespective of transcriptional status. Recent epigenomic profiling studies have begun to reveal a significant portion of CGIs that are subject to varying degrees of tissue-specific methylation in human somatic tissues [40]–[42]. These findings point to the existence of processes that protect CGIs from *de novo* methylation, which can be selectively inactivated during development and may become defective during cancer progression [43]. Definition of the *cis*-regulatory elements and *trans*-acting factors that control CGI methylation status is key to unraveling these processes.

We have revisited the well established example of the *APRT* gene CGI promoter. It was previously shown that SP1-like binding elements are required to prevent CGI methylation [28],[29], although surprisingly the SP1 transcription factor itself is not required [30]. These findings suggested that other factors function at the G-rich SP1-like motifs, which are commonly found at CpG islands [44]. We show that VEZF1 interacts with site 3 of the hamster *APRT* CGI. A promoter-less *APRT* CGI fragment containing only site 3 remains protected from DNA methylation [28]. We were able to abrogate SP factor binding while retaining VEZF1 binding by introducing VEZF1-specific elements. The VEZF1-specific mutant retained its ability to mediate demethylation and protection from *de novo* DNA methylation. Thus, VEZF1 binding elements can protect a CGI from DNA methylation. We attempted to definitively address the requirement for VEZF1, but discovered that global *de novo* DNA methylation mechanisms are defective in *Vezf1* null ES cells [27]. We also show that VEZF1 interacts with the CGI promoter of the *DHFR* gene (Figure S5). Furthermore, ChIP-array analysis in somatic human cells reveals that VEZF1 predominantly interacts with CGI promoters and regulates genes with diverse functions (R.S. and A.W., unpublished observations). It remains to be determined whether VEZF1 plays a widespread role in the control of DNA methylation and what contribution this epigenetic control makes to developmental gene regulation and cancer progression.

### DNA methylation and the multiple roles of chromatin boundaries

Experimental evidence has demonstrated that chromatin barrier elements can employ a number of different mechanisms to limit the spread of transcriptionally repressive chromatin; including tethering, nucleosome gaps/masking and histone code manipulation [6]. The constitutive recruitment of histone modifications such as acetylation is considered to be sufficient to establish barrier activity in eukaryotes that do not methylate their genomes, as demonstrated at synthetic barriers in yeast [45]. Our finding that HS4 also mediates protection from *de novo* DNA methylation adds another tier to understanding the mechanism of barrier elements in vertebrates. It is well established that densely methylated DNA abrogates transcription factor binding and is sufficient to establish all the features of repressive chromatin, including repressive histone modifications [46],[47]. We propose that a barrier element in higher eukaryotes must be capable of preventing *de novo* DNA methylation in addition to blocking the propagation of silencing histone modifications, as either event, if not inhibited, is sufficient to direct the establishment of an epigenetically stable silent chromatin state. We have shown here that a fully effective vertebrate barrier combines both of these properties in a single multi-component element.

## Materials and Methods

### Bisulfite sequencing analysis

Chicken 6C2 erythroleukemia cells carrying *IL-2R* reporter transgenes (8103, wild type HS4; 10401, ΔFI; 10506, ΔFII; 10615, ΔFIII; 10901, ΔFIV; 8d5, ΔFV) were cultured and assayed for IL-2R expression by FACS as described previously [12],[14]. Genomic DNA was extracted from cell lines after 30 and 90 days of culture and bisulfite modified (EZ Methylation, Zymo Research, CA). The upstream double-core HS4 elements and the IL-2R promoter were PCR amplified from each line. We were unable to amplify bisulfite modified double-core HS4 elements from the 10506, ΔFII line to sufficient levels to provide representative sequence data. We therefore opted to amplify the outermost HS4 copy only. The 8d5, ΔFV line also only contains one copy of HS4 in the upstream location [12]. All PCR products were gel purified and cloned, followed by sequencing (GATC Biotech, Konstanz, Germany) of 10 clones for each region of interest.

*Gel mobility shift assays* were performed as described previously [14]. Recombinant VEZF1, SP1, SP3 and ZF5 were produced by *in vitro* translation using rabbit reticulocyte lysate (Promega).

### Protein purification

FI- and FIII-binding proteins were purified from adult chicken red blood nuclear protein extracts by ion exchange chromatography. Throughout the purification, eluate fractions were analyzed for FI- and FIII-binding activity with gel mobility shift assays. The binding specificity of partially purified proteins was checked by competition analysis after each purification step. FI- and FIII-binding activities co-fractionated following ion exchange chromatography with SP XL and Heparin sepharose (GE Healthcare). Phosphocellulose, Q and Sephacryl S300 columns were used in early purification attempts to resolve FI- and FIII-binding activities but they co-fractionated in each case (data not shown). FI- and FIII-binding activities both eluted in two distinct fractions of approximately 200 and 400 kDa following gel filtration (data not shown). The active fractions eluted from heparin sepharose were pooled then split into two and fractionated in parallel by FI or FIII DNA affinity as described previously [14]. Polypeptides electrophoresed on 7% Tris-acetate gels (Invitrogen) were excised, digested with trypsin and sequenced at the Harvard Microchemistry Facility by microcapillary reverse-phase HPLC nano-electrospray tandem mass spectrometry (μLC/MS/MS) on a Finnigan LCQ DECA quadrupole ion trap mass spectrometer.

### cDNA cloning

Chicken VEZF1/BGP1 cDNA was cloned following RT-PCR from 6C2 cell total RNA based on an assumption of conservation of 5′ cDNA sequence with human VEZF1/DB1. The oligonucleotide Adaptor-A 5′CATGCCGCTCGAGCGGTTTTTTTTTTTTTTTTT was used in first strand cDNA synthesis with Superscript II reverse transcriptase (Invitrogen). The primers VEZF1_5′, 5′CCATGACCCATGGGCAGAGCCAAAGT and Adaptor 5′CATGCCGCTCGAGCGG were used to amplify a full length chicken VEZF1 cDNA by PCR which was TA cloned into pCRII (Invitrogen). VEZF1 cDNA was sub-cloned into pCITE4b (Novagen) to generate p4bVEZFfull for the purpose of *in vitro* transcription. cDNA encoding chicken ZF5 was isolated by RT-PCR from 6C2 cell total RNA using primers designed from the published sequence (U51640). We found that the bases CpG 1306-7 in the published sequence were GpC in our clone, causing codon 436 to translate as alanine instead of arginine. We obtained the vectors pCDNA3-ZF5 and pEVRFO-ZF5 (a kind gift of W. Stumph, San Diego State University) and we also found the CpG to GpC conflict with the published sequence. Full length chicken SP1 and SP3 cDNAs cloned in the pBluescript-based vectors pH-SP1 and pH-SP3-3 were a kind gift from Marc Castellazzi (INSERM, Lyon).

### Antibodies

Polyclonal antibodies were raised (Rockland Immunochemicals) against chicken VEZF1 (Ser376-Ala547) and chicken ZF5 peptides (Ser131-Lys248) produced in *E.coli* (QIAexpress, Qiagen). Peptides were produced in M15 [pREP4] *E. coli* followed by rapid lysis with B-PER reagent (Pierce). ZF5 peptide was soluble and purified on Ni-NTA agarose (Qiagen). VEZF1 peptide was insoluble and resulting inclusion bodies were prepared using B-PER reagent (Pierce), solubilized with 6M guanidium hydrochloride and immobilized on TALON Sepharose resin (Clontech) at pH 7. VEZF1 peptide was renatured in a stepwise manner with 6, 4, 3, 2, 1 and 0.5 M guanidium hydrochloride prior to elution. VEZF1 and ZF5 polyclonal IgG antibodies (Rockland Immunochemicals) were purified from rabbit serum using PROSEP-A media (Montage, Millipore). Anti-full length chicken VEZF1 antibodies were raised previously [27]. Antibodies raised against CTCF (06-917), SP1 (PEP2X, H-225X), SP3 (D20X) and USF1(B01) were obtained from Millipore, Santa Cruz Biotechnology and Abnova, respectively.

### Chromatin immunoprecipitation (ChIP) analysis

ChIP analysis of transcription factor binding in chicken cells was performed using formaldehyde crosslinked chromatin prepared from chicken 10 day embryonic erythrocytes isolated from fertilized White Leghorn eggs (CBT Farms, Chestertown, MD) or cultured 6C2 erythroleukemia cells. 10 day erythrocytes were washed and resuspended in 25 mls of PBS (2×10^7^ cells/ml) and fixed with a final concentration of 0.25% formaldehyde at room temperature for 30 seconds. 6C2 cells were harvested in mid-exponential growth phase, divided into 30 ml aliquots containing 1×10^8^ cells in fresh media and fixed with a final concentration of 0.8% formaldehyde at room temperature for 5 minutes. Reactions were stopped by adding glycine to a final concentration of 0.125 M. The cells were washed in PBS and resuspended in cell lysis buffer (0.25% Triton X-100, 10 mM EDTA, 0.5 mM EGTA, 10 mM Tris pH 8.0) to isolate nuclei. Chromatin was prepared following washing (0.2 M NaCl, 1 mM EDTA, 0.5 mM EGTA, 10 mM Tris pH 8.0) and lysis (NLB: 50 mM Tris-HCl pH 8.0, 10 mM EDTA, 0.5% SDS) of nuclei. Crosslinked chromatin was fragmented by sonicaton (Misonix) for a total time of 10 minutes in regular 10 second pulses at 4°C. Debris was removed by centrifugation at 15000 g for 10 minutes and chromatin was diluted in 10 volumes of X-ChIP buffer (1.1% TX-100, 1.2 mM EDTA, 16.7 mM Tris pH 8.1, 167 mM NaCl). Agarose gel electrophoresis was used to confirm that chromatin fragments were ∼500 bp in length on average.

Chromatin was pre-cleared with 100 µl of protein A agarose (50% slurry in X-ChIP buffer, Millipore) and 50 µg of normal rabbit IgG (Santa Cruz) for 3 hours at 4°C on a rotating wheel. Aliquots of chromatin were taken to generate input DNA and protein for western analysis. Individual ChIPs were performed using chromatin from 1×10^7^ cells in a total volume of 1 ml by diluting the pre-cleared chromatin with modified X-ChIP buffer (1 part NLB to 9 parts X-ChIP). Incubation with antibodies was performed overnight at 4°C on a rotating wheel. Between 10 and 30 µg of specific antibodies or 10 µg of normal rabbit IgG (Santa Cruz) were used per ChIP. Chromatin was precipitated with protein A agarose (50 µl slurry in X-ChIP, Millipore) for 4 hours at 4°C with rotation. Immunoprecipitated chromatin was collected, washed, eluted and crosslinks reversed. DNA was then extracted by phenol-chloroform and ethanol precipitated in the presence of 10 µg glycogen.

Relative DNA enrichments were quantified by TaqMan real-time qPCR using the comparative Ct method relative to input DNA and normalized to the primer set 10.35 within the 16 kb condensed chromatin region upstream of the chicken *β-globin* locus as described previously [14]. The TaqMan primer sets 10.35 (“16 kb”), 21.54 (HS4 core, “end HS4”), 39.806 (beta-adult promoter), 50.861 (3′HS) and PGI5′ (5′ HS4 elements on IL-2R transgene, “trans HS4”) were used in this study.

10.35_For: GGAACAAGTTGGCAAGGTCCTAT

10.35_Rev: TCTTCTGCCCTGCCCGTAT

10.35_TM: FAM-TGCAGTTCCCTGTTCATGTGCTTTTCG-TAMRA

21.54_For: TCCTGGAAGGTCCTGGAAG

21.54_Rev: CGGGGGAGGGACGTAAT

21.54_TM: 6FAM-CCCAAAGCCCCCAGGGATGT-TAMRA

39.8_For: CTGTGGTCTCCTGCCTCACA

39.8_Rev: AGGCTGGGTGCCCCTC

39.8_TM: FAM-CAATGCAGAGTGCTGTGGTTTGGAACTG-TAMRA

PGI_5′_For CACAGGAAACAGCTATGACATGATT

PGI_5′_Rev TCTGCCTTCTCCCTGATAACG

PGI_5′_TM 6FAM-AATTCCTGCCCACACCCTCCTGC-TAMRA

ChIP analysis of transcription factor binding in murine E14Tg2A.4 ES cell lines was performed using formaldehyde crosslinked chromatin prepared from 1×10^9^ cells treated with 1% formaldehyde for 5 minutes. Chromatin was prepared as described above, where fragments sizes ranged from 500–700 bp. Semi-quantitative PCR was performed using the following primers

APRT1/2_For: AAAGGCGTGCGGGAGCCAGAAAT

APRT1/2_Rev: CCTTGGTAGGTGGGG

APRT3_For: CCCTGTTCCTGGGCTCC

APRT3_Rev: TGACTGGCCAGGAGG

ChIP analysis from human embryonic kidney 293-T cell line SD5 that contains a stably integrated copy of the 275 bp HS4 core chicken insulator was performed as described for cultured chicken cells above. SD5 cells were crosslinked with 1.6% formaldehyde for 5 minutes. The following primers were used in SYBR quantitative PCR analysis:

HS4_21.726_F: CGGGATCGCTTTCCTCTGA

HS4_21.726_R: CCGTATCCCCCAGGTGTCT

P_DHFR_F: TCGCCTGCACAAATAGGGAC

P_DHFR_R: AGAACGCGCGGTCAAGTTT

Control

VEZF1_CDS_F: GACAGCAGCCGAACTTCGTT

VEZF1_CDS_R: TGGTGCCCGAGGAAGATG

*APRT elements.* The following elements were amplified from Hamster liver genomic DNA:

#### Wild-type *APRT* CpG island

Sp1-like motifs in red. *Ava*II and *Hpa*II restriction sites in bold.

CTAGAGGATCCGGACAACACCCACACCGGCCCCTCCAGGTCCAGAAAGCTGGCCCTGCGAGAAGCGGGACTGAAAAGGCGTGCGGGAGCCAGAAATCCAAAAGGGTGCCAAGGCATGCGTCCTTTTTCCACCCAGAAATAACCCCAGGCTTTCAATTTGAGGTTATTTCAATATCCAGCAAATGCGTTACTTCCTGCCAAAAGCCAGCCTCCCCGCAACCCACTCTCCCAGAGGCCCCGCCCCGTCCCGCCCCCTCCCGGCCTCTCCTCGTGCTGGATCGCTCCCTAAGGACGCCCCGCTCCAGAAGCCCCACCTACCAAGGACGCCCCACCCTTGTTCCCGGACTGGTATGACCCCAGCCTGCTGACATCCCTCCGCCCTTTCTCGTGCACGCGGCTATGGCGGAATCTGAGTTGCAGCTGGTGGCGCAGCGATCCGCAGTTTCCCCGACTTCCCCATCCCCGGCGTGCTGTTTAGGTGAGATCACGAGCCAGCAAGGCGTTGGAGCCCTGTTCCTGGGCTCCCGGCGAGGCGCATGGGCAGTCTCGGGGATCTTGTGGGGTCTCCGCCCCCCTTTCCCCGGCCACCAGCCTCTCCTTGTTCCCAGGGATATCTCGCCCCTCCTGAAGGACCCCGCCTCCTTCCGAGCTTCCATCCGCCTCCTGGCCAGTCACCTTAAGTCCACGCATGGCGGCAAGATCGACTACATCGCAGGTCTA

#### Mutant *APRT* CpG island

Mutated bases in blue were introduced by site directed mutagenesis. Overall CpG content increased from 46 to 48 following these mutations. *Ava*II and *Hpa*II restriction sites in bold.

CTAGAGGATCCGGACAACACCCACACCGGCCCCTCCAGGTCCAGAAAGCTGGCCCTGCGAGAAGCGGGACTGAAAAGGCGTGCGGGAGCCAGAAATCCAAAAGGGTGCCAAGGCATGCGTCCTTTTTCCACCCAGAAATAACCCCAGGCTTTCAATTTGAGGTTATTTCAATATCCAGCAAATGCGTTACTTCCTGCCAAAAGCCAGCCTCCCCGCAACCCACTCTCCCAGAGACCCCCCGCATCCCCGACGCTACCCCCCGCATCCCCGATCTCCTCGTGCTGGATCGCTCCCTAAGGACGCCCCGCTCCAGAAGCCCCACCTACCAAGGACGCCCCACCCTTGTTCCCGGACTGGTATGACCCCAGCCTGCTGACATCCCTCCGCCCTTTCTCGTGCACGCGGCTATGGCGGAATCTGAGTTGCAGCTGGTGGCGCAGCGATCCGCAGTTTCCCCGACTTCCCCATCCCCGGCGTGCTGTTTAGGTGAGATCACGAGCCAGCAAGGCGTTGGAGCCCTGTTCCTGGGCTCCCGGCGAGGCGCATGGGCAGTCTCGGGGATCTTGTGGGGTCCGCTCCCCCCGCATCCCCGACACCAGCCTCTCCTTGTTCCCAGGGATATCTCGCCCCTCCTGAAGGACCCCGCCTCCTTCCGAGCTTCCATCCGCCTCCTGGCCAGTCACCTTAAGTCCACGCATGGCGGCAAGATCGACTACATCGCAGGTCTA

#### Non-CpG island from *APRT* locus

*Hpa*II site in bold.

TCTAGATTGCTAGGAGTAGCACCTAAGATGAACTAGATGCTAAAAAATGCTGTATCTTTGGGGCACACGAGGGCATGCCTGGGCAGGCTTAGAGCCTGGTAGTCTCAGGGGCTGCACCAAAGTGTAATTCTTGTGCTAAATAACTTTCACTTACCAGTGCCAAGCACGGGCTTCAGAAACACCCTAGGGTCGCTGAATGTCCACCAGGGGAGTCAGACATGTCCAGAGGGTGAGAACCCCAGAGAATTCGGTAGCCCTGACATGTGCTACAATTACTGATGCCCACTTCCTACTGGTTCCTCCTGGCCATACCTCAGGAATTAGGGCATGCTTTCTGCCTGCTACAGTAGCTCATCCTCCCTGGAAGTGACCCCAGACATATACCCTGAACTGTAACCGATAAAGTGCGCCTGGGCAGATGTATTTGAGAGGTGGCAAAAGTAAACCATAGGTGTCCCCGAGCTAGATACAGAAGGCAGATAACATCCCCAAGGCTAAGCTGCTGCCCCAATAGCCATCAGCCTTCTAGTTATAGCTAGTAAGACCTAGTATTCCTGGTCAATACTATTCACTCAATCCTTACACCTCAGCCCTAACACGCCCCCTCTCTCATCCTAACAGGCCTAGACTCCAGGGGATTCTTGTTTGGCCCCTCCCTAGCTCAGGAGCTGGGCCTGGGCTGTGTGCTCATCCGGAAGCGAGGGAAGCTGCCAGGCCCCACAGTGTCAGCCTCCTATGCTCTCGAGTATGGCAAGGTAAGCAGGCAGTGGGTAGCTGTCTAGGAGTAAATGTGGGGGCTCAGAGAGGTTAAGTCATCAGGCCAGGTTTATACCACCAGGAAACATGGAGAAGCTAGGGGTGGTGGTTCTAGA

### CpG island methylation assays

∼720 bp wild type and mutant *APRT* CpG islands and an 870 bp non-island region of the *APRT* gene were cloned into pUC19. Each element was PCR amplified, half of which was subject to *in vitro* CpG methylation by M.*Sss*I methyltransferase(New England Biolabs). *In vitro* methylation was validated by *Hpa*II and *Msp*I digestion. 1 µg of each *APRT* element was transfected into murine E14Tg2A.4 ES cells (BayGenomics) with 1 µg of the *Xba*I fragment of pREP7 (Invitrogen). Hygromycin resistant clones were grown in LIF without feeder co-culture. Genomic DNA was extracted after two weeks of culture. Probes for Southern blotting were generated using Ready-to-go dCTP beads (GE Healthcare).

### Oligonucleotide sequences

Oligonucleotides were generated on an ABI 394 DNA synthesizer.

The top strand sequences for each of the duplexes used for gel mobility shift analyses were:

FI wt 5′ GGAGCTCACGGGGACAGCCCCCCCCCAAAGCCCCCAGGGA,

FIII wt 5′ aggcgcgccCCGGTCCGGCGCTCCCCCCGCATCCCCGAGCCGGggcgcgcct,

FV wt 5′ CCTGCAGACACCTGGGGGGATACGGGGAAAAAGCTTTAGG,

Sp1 5′ ATTCGATCGGGGCGGGGCGAGC,

glo wt 5′ AATTGCAGAGCTGGGAATCGGGGGGGGGGGGGGGGCGGGTGGTGGTGTGG,

glo 7G 5′ AATTGCAGAGCTGGGAATCGGGGGGGCGGGTGGTGGTGTGG,

glo 6G 5′ AATTGCAGAGCTGGGAATCGGGGGGCGGGTGGTGGTGTGG.

*Asc* I restriction sites used for cloning FIII sites in an earlier study are shown as lower case. All FI and FIII oligonucleotides were identical to the wt sequences above except for mutations indicated in Figure 1E.

DNA affinity columns were prepared using the following oligonucleotides

FI-DA TOP 5′ gatcTCACGGGGACAGCCCCCCCCCAAAGCCCCCA

FI-DA BOTTOM 5′ gatcTGGGGGCTTTGGGGGGGGGCTGTCCCCGTGA

FIII-DA TOP 5′ gatcGGTCCGGCGCTCCCCCCGCATCCCCGAGCCGGCA

FIII-DA BOTTOM 5′ gatcTGCCGGCTCGGGGATGCGGGGGGAGCGCCGGACC

PCR primers used in bisulfite sequencing were

HS4 5′ double forward 5′ GGTATTAGAGTAGATTGTATTGAGAGTGTA

HS4 5′ double reverse 5′ CATAACTATTTCCTATATAAATCCCC

HS4 5′ single forward 5′ AGAGTAGATTGTATTGAGAGTGTATTATA

HS4 5′ single reverse 5′ ACATCCCTAAAAACTTTAAAAAAAA

IL-2R forward 5′ GTTAAGGTTGGGGGTTTTTT

IL-2R reverse 5′ AAAACTCTACCTAACAACCAAACAC

PCR primers used in RT-PCR gene expression analysis were

GgVEZF805anti 5′CAGTGCACGTTTGGCATTTGAAG

GgVEZF716sense 5′GAAAAGGCTTCTCGAGGCCTGATC

GgGAPDH-247T 5′-6FAM-TCCAGGAGCGTGACCCCAGCA-TAMRA

GgGAPDH-226F 5′ ATGGGCACGCCATCACTATC

GgGAPDH-302R 5′ AACATACTCAGCACCTGCATCTG

GgB-ACTIN_F 5′ TGCTGCGCTCGTTGTTGA

GgB-ACTIN_R 5′ CATCGTCCCCGGCGA

GgB-ACTIN_T 5′-6FAM-TGGCTCCGGTATGTGCAAGGCC-TAMRA

The primers used for methylation specific PCR analysis of the *APRT* non-island element were:

APRTNI_5′ TCTAGATTGCTAGGAGTAGC

APRTNI_3′ TCTAGAACCACCCCTAGC

## Supporting Information

Figure S1 Core HS4 sequences. (A) The 244 bp *Sac*I - *Hin*dIII fragment originally defined as the “250 bp core” HS4 element [11]. Underlined bases indicate the positions of five *in vitro* DNaseI footprints [11]. Bases in bold type have been shown to be essential for DNA-binding of CTCF [9], USF1/2 [14], or VEZF1 (this study). This 244 bp fragment alone has not been tested in either enhancer blocking or barrier assays. (B) The 275 bp HS4 fragment used to define insulator properties of the “250 bp core” in enhancer blocking [9] and barrier assays [12]. Flanking *Asc*I cloning sites present in all functional assays of the HS4 core are shown in italics.
(0.87 MB TIF)

Figure S2 Identification of FI- and FIII-binding proteins. (A) Peptide sequences obtained from tandem MS sequencing of proteins isolated by FI- and FIII-DNA affinity. (B) Alignment of the amino acid sequences of chicken VEZF1/BGP1, human VEZF1/DB1, and mouse Vezf1 (accession numbers AY775302, 1082846, and 7710108). Peptides obtained from tandem MS sequencing that match VEZF1 are indicated by lines above the alignment. Six C2H2 zinc fingers motifs are boxed. (C) Schematic representation of the domain structure of VEZF1. Only the zinc finger motifs share homology with factors other than VEZF1 orthologs, the nearest relative being the MAZ transcription factor.
(4.96 MB TIF)

Figure S3 Supershift analysis of VEZF1, SP1, SP3, and ZF5 interactions with HS4 footprints and the *β^A^-globin* promoter. Gel mobility supershift assays using ^32^P-labelled FI (A), FIII (B), FV (C), and glo wt (D) oligonucleotide duplexes. Adult chicken erythrocyte nuclear extract (ery) and recombinant chicken VEZF1, SP1, or SP3 used in the reactions are indicated by brackets above the lanes. Proteins were pre-incubated with antibodies (indicated above each lane) prior to incubation with DNA. Supershifts are evidenced by abrogation of specific complexes (asterisks) and/or formation of low mobility ternary complexes (SS). Antibodies alone do not give rise to complexes with any of the duplexes used (not shown).
(3.49 MB TIF)

Figure S4 Analysis of SP1, SP3, and ZF5 interactions with HS4 footprints I and III. Gel mobility shift assays using ^32^P-labelled FI and FIII oligonucleotide duplexes. Unlabelled competitor duplexes (indicated above each lane) were added at 50 fold molar excess. Recombinant chicken SP1, SP3, and ZF5 used in the reactions are indicated by brackets above the lanes. The competition profile of these proteins does not match that of red blood cell nuclear extract (Figure 4) or recombinant VEZF1 (Figure 6A).
(0.71 MB TIF)

Figure S5 SP1, SP3, and ZF5 do not interact with HS4 *in vivo*. (A) ChIP analysis of transcription factor interactions at the *β-globin* locus in 6C2 erythroid progenitor cells. DNA enrichments at the {lower case beta}A promoter or the HS4 and 3′HS insulators were normalized to a negative control located in the 16 kb condensed chromatin region upstream of the *β-globin* locus. (B) ChIP analysis of transcription factor interactions with a stably integrated HS4 element in transgenic human 293 cells. Interactions with the *DHFR* CpG island promoter are shown as a positive control for SP1 and SP3 binding. DNA enrichments are normalized to an AT-rich negative control locus.
(0.49 MB TIF)

Figure S6 VEZF1 binding to HS4 is resistant to individual binding site mutations. (A) Schematic representation of the *IL-2R* transgene showing location of QPCR primer sets used to analyze the interaction of VEZF1 with the transgenic HS4 and promoter elements. (B–D) ChIP analysis of (B) VEZF1, (C) CTCF, or (D) USF1 interactions at the endogenous HS4 insulator (eHS4) and the transgenic HS4 (tHS4) and promoter (β-IL2Rpro) elements in the same 6C2 cell lines used for DNA methylation (Figure 3) and histone modification [14] analysis. DNA enrichments were normalized to a negative control located in the 16 kb condensed chromatin region upstream of the *β-globin* locus. Deletion of either footprint II or footprint IV disrupts CTCF and USF1 binding as expected. In contrast, deletion of individual VEZF1 sites footprint I, III, or V have no significant effect on overall VEZF1 ChIP efficiency.
(1.09 MB TIF)

Figure S7 VEZF1 binding to HS4 is not significantly affected following VEZF1 RNAi. (A) Quantitative RT-PCR analysis following 48 hours of doxycycline-induction of two chicken 6C2 cell lines harboring lentiviral vectors that express VEZF1-specific miRNA. Expression levels are normalized to those of β-actin (ACTB) and untreated cells. VEZF1 mRNA levels are reproducibly knocked down by 70%. (B) Western blot analysis of chicken VEZF1 protein levels in one of the above lines with and without 14 days of doxycycline induction of VEZF1-specific miRNA. TBP levels were monitored as a loading control. VEZF1 and TBP band intensities visualized on a FUJI LAS3000 imager were quantified using AIDA software (shown below). Following normalization to TBP levels, VEZF1 protein levels were quantified to be knocked down by ∼97%. VEZF1-specific miRNA may cause translational inhibition in addition to the mRNA degradation observed by RT-PCR (C) ChIP analysis of VEZF1 interaction with the endogenous HS4 element following 14 days of induced VEZF1 knockdown. DNA enrichments were normalized to a negative control located in the 16 kb condensed chromatin region upstream of the *β-globin* locus.
(1.64 MB TIF)

Figure S8 Transcription factor interactions with *APRT* CpG island elements. VEZF1 and SP1 interact with the wild type *APRT* CpG island, but the FIII mutant *APRT* element is only bound by VEZF1. Gel mobility supershift assays using ^32^P-labelled oligonucleotide duplexes containing either the wild type *APRT* SP1 sites 1&2 (lanes 1–4) and site 3 (lanes 5–8) or the mutant sites 1&2 (lanes 9–12) and site 3 (lanes 13–16). The core sequences of the duplexes are as shown in figure 8a. Nuclear extracts were pre-incubated with antibodies (indicated above each lane) prior to incubation with DNA. Supershifts are evidenced by the formation of low mobility ternary complexes (SS) in addition to abrogation of specific complexes (asterisk). Antibodies alone do not give rise to complexes with any of the duplexes used (not shown). SP1 and SP3 are detected in complexes with the wild type sites 1&2 and site 3 only. VEZF1 is detected in complexes with all four sites; weakest binding is seen at wild type sites 1&2 and strongest binding is seen at mutant sites 1&2.
(1.78 MB TIF)

Table S1 VEZF1 sites in the HS4 barrier protect a promoter from DNA methylation. CpG methylation of transgene promoters flanked by wild type or mutant HS4 insulators after 30 or 90 days of culture. The scoring of individual CpG bases from each clone subject to bisulfite sequencing is shown. Methylated bases are marked as ‘1’ and shaded blue. Average CpG methylation values are shown in Figure 5. Numbers above each table refer to CpG numbering from [16], where CpG 4–11 and 12–18 reside in the promoter and coding sequence, respectively.
(0.02 MB PDF)

Table S2 Mutations of insulator protein binding sites result in the *de novo* methylation of HS4. CpG methylation of wild type (WT) or mutant (ΔI - ΔV) HS4 insulators after 30 or 90 days of culture. Both copies of HS4 were sequenced, except for ΔII and ΔV, where only the outermost copy was sequenced (see Materials and Methods). The scoring of individual CpG bases from each clone subject to bisulfite sequencing is shown. Methylated bases are marked as ‘1’ and shaded blue. Average CpG methylation values are shown in Figure 3. Numbers above each table refer to CpG numbering as assigned in Figure 3b.
(0.03 MB PDF)

Text S1 DNA binding activities of candidate HS4-binding proteins; RNA interference methods; western blotting methods.
(0.04 MB DOC)
