# Supplementary figures and images for: VEZF1 Elements Mediate Protection from DNA Methylation
Source: PLoS Genet. 2010 Jan 8;6(1):e1000804. doi: 10.1371/journal.pgen.1000804 (PMC2795164; doi:10.1371/journal.pgen.1000804)

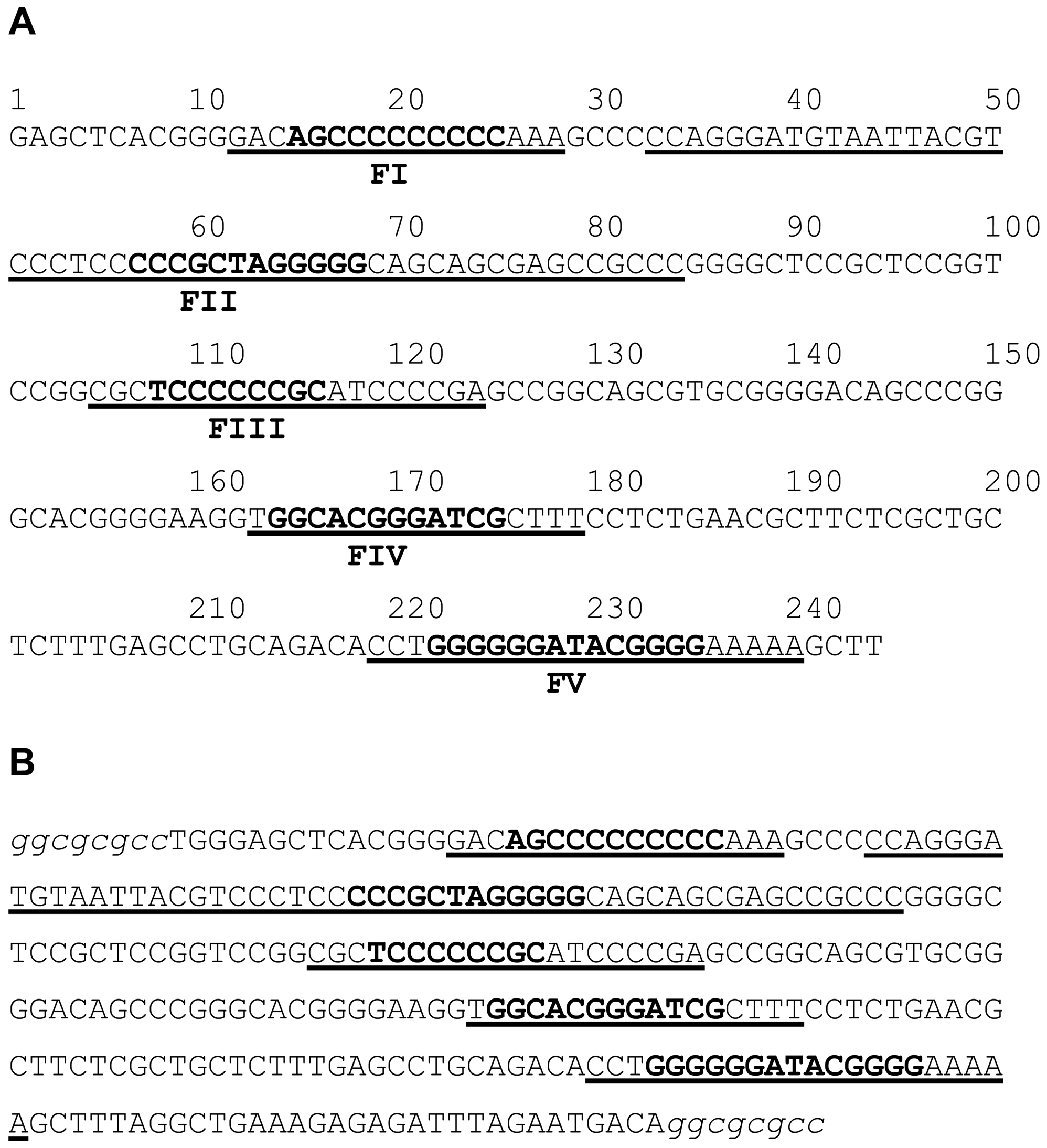

Supplement: Figure S1 — Core HS4 sequences. (A) The 244 bp SacI - HindIII fragment originally defined as the “250 bp core” HS4 element [11]. Underlined bases indicate the positions of five in vitro DNaseI footprints [11]. Bases in bold type have been shown to be essential for DNA-binding of CTCF [9], USF1/2 [14], or VEZF1 (this study). This 244 bp fragment alone has not been tested in either enhancer blocking or barrier assays. (B) The 275 bp HS4 fragment used to define insulator properties of the “250 bp core” in enhancer blocking [9] and barrier assays [12]. Flanking AscI cloning sites present in all functional assays of the HS4 core are shown in italics. (0.87 MB TIF) [file pgen.1000804.s001.tif]

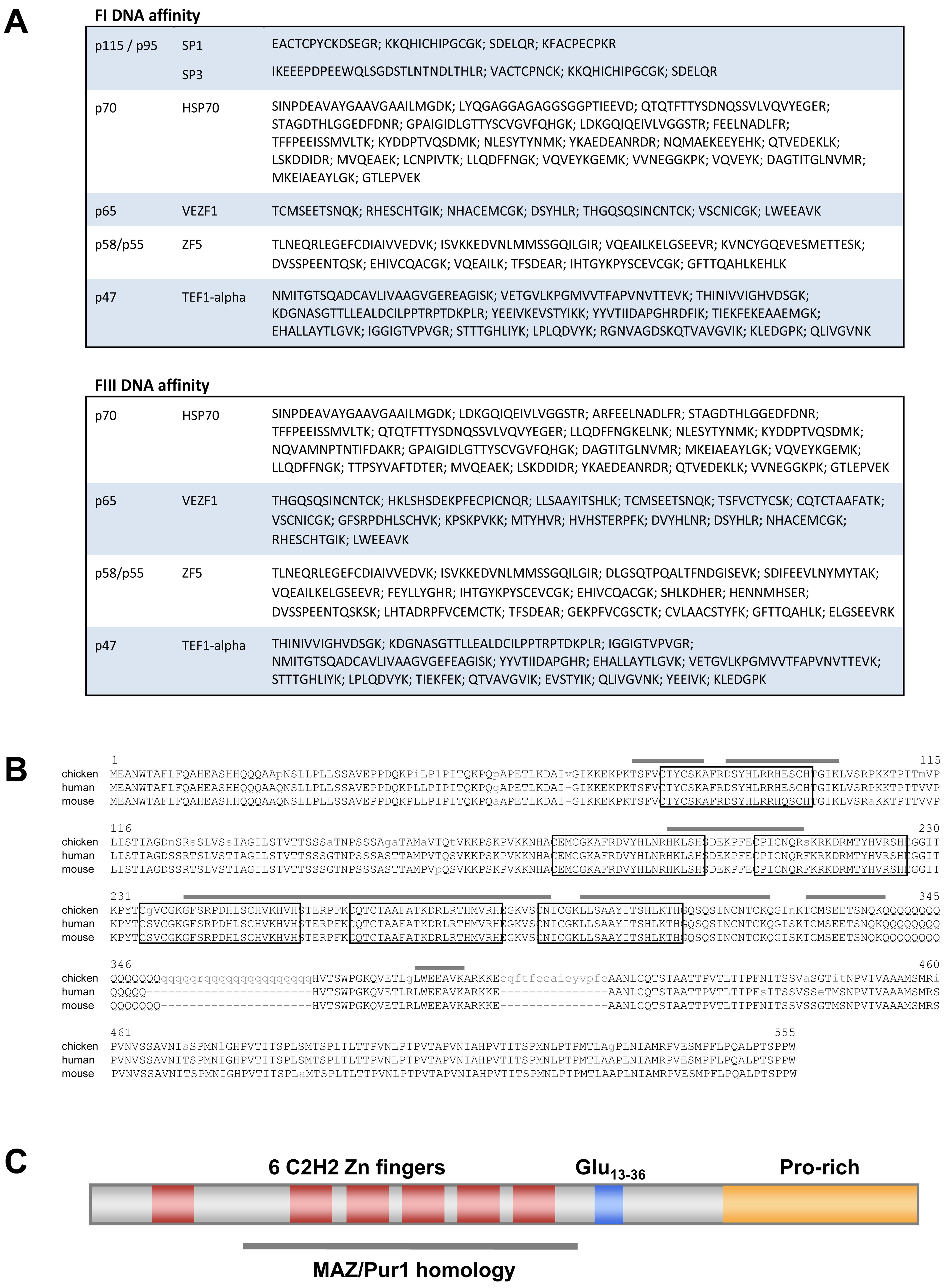

Supplement: Figure S2 — Identification of FI- and FIII-binding proteins. (A) Peptide sequences obtained from tandem MS sequencing of proteins isolated by FI- and FIII-DNA affinity. (B) Alignment of the amino acid sequences of chicken VEZF1/BGP1, human VEZF1/DB1, and mouse Vezf1 (accession numbers AY775302, 1082846, and 7710108). Peptides obtained from tandem MS sequencing that match VEZF1 are indicated by lines above the alignment. Six C2H2 zinc fingers motifs are boxed. (C) Schematic representation of the domain structure of VEZF1. Only the zinc finger motifs share homology with factors other than VEZF1 orthologs, the nearest relative being the MAZ transcription factor. (4.96 MB TIF) [file pgen.1000804.s002.tif]

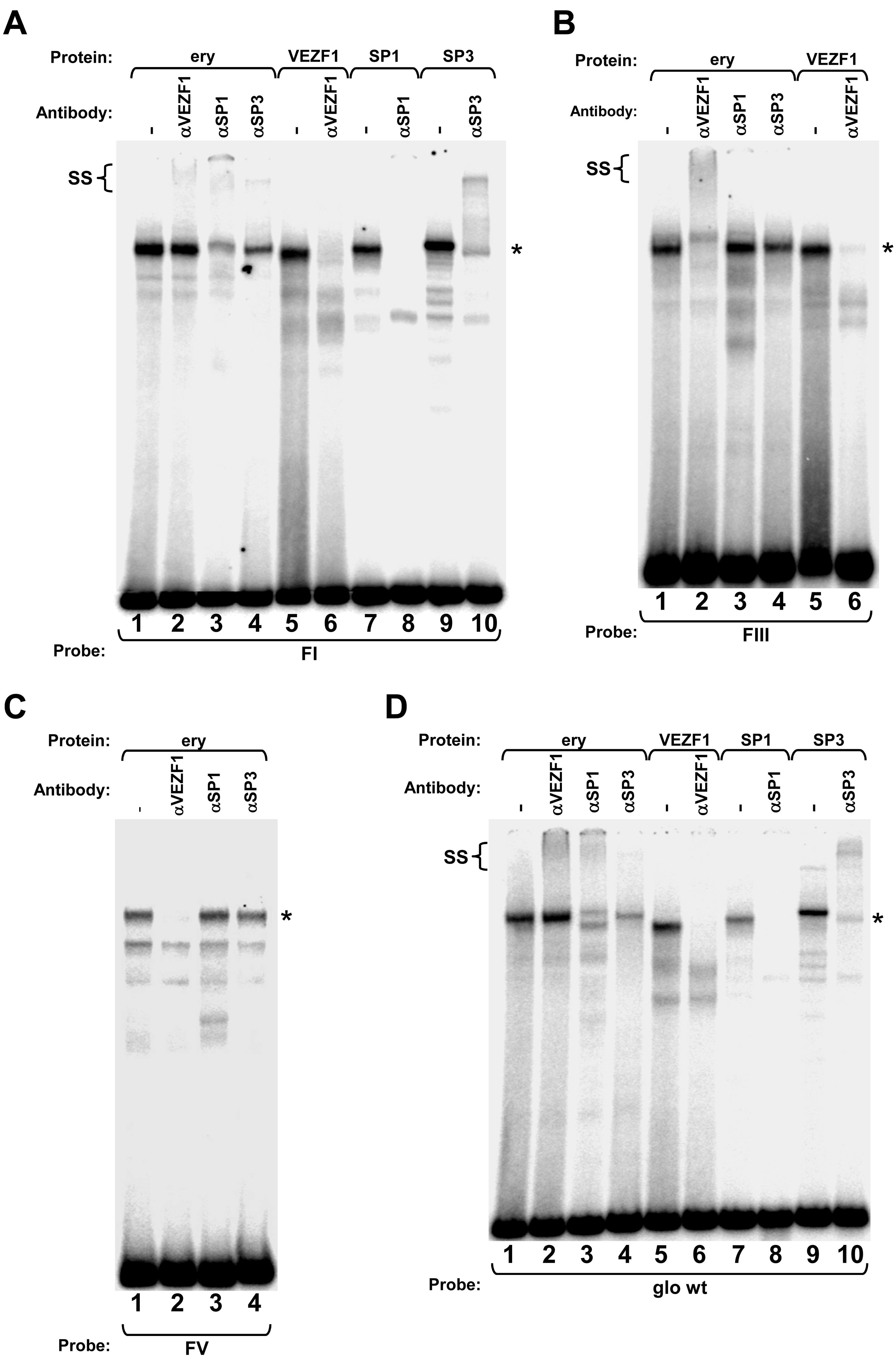

Supplement: Figure S3 — Supershift analysis of VEZF1, SP1, SP3, and ZF5 interactions with HS4 footprints and the βA-globin promoter. Gel mobility supershift assays using 32P-labelled FI (A), FIII (B), FV (C), and glo wt (D) oligonucleotide duplexes. Adult chicken erythrocyte nuclear extract (ery) and recombinant chicken VEZF1, SP1, or SP3 used in the reactions are indicated by brackets above the lanes. Proteins were pre-incubated with antibodies (indicated above each lane) prior to incubation with DNA. Supershifts are evidenced by abrogation of specific complexes (asterisks) and/or formation of low mobility ternary complexes (SS). Antibodies alone do not give rise to complexes with any of the duplexes used (not shown). (3.49 MB TIF) [file pgen.1000804.s003.tif]

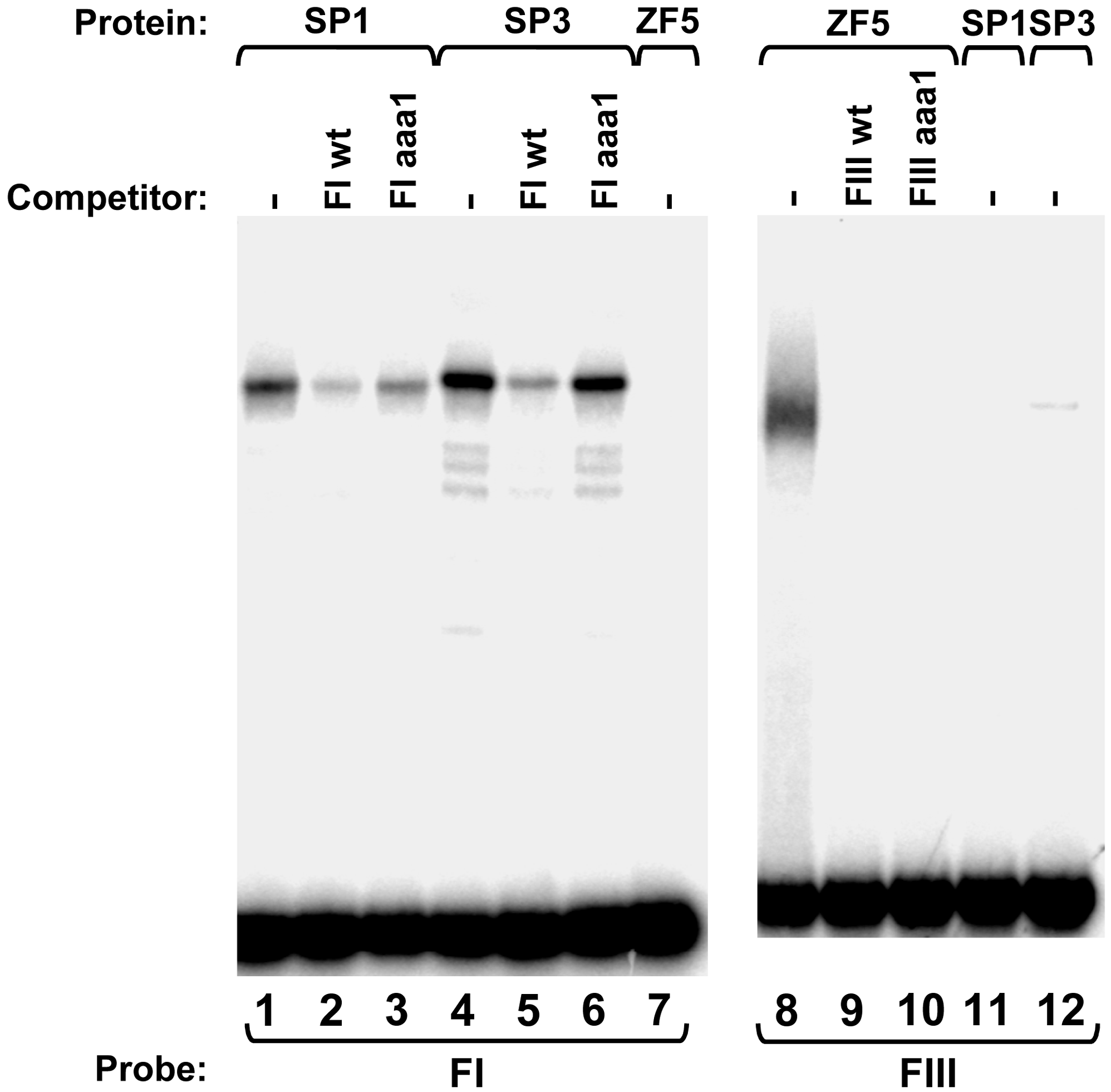

Supplement: Figure S4 — Analysis of SP1, SP3, and ZF5 interactions with HS4 footprints I and III. Gel mobility shift assays using 32P-labelled FI and FIII oligonucleotide duplexes. Unlabelled competitor duplexes (indicated above each lane) were added at 50 fold molar excess. Recombinant chicken SP1, SP3, and ZF5 used in the reactions are indicated by brackets above the lanes. The competition profile of these proteins does not match that of red blood cell nuclear extract (Figure 4) or recombinant VEZF1 (Figure 6A). (0.71 MB TIF) [file pgen.1000804.s004.tif]

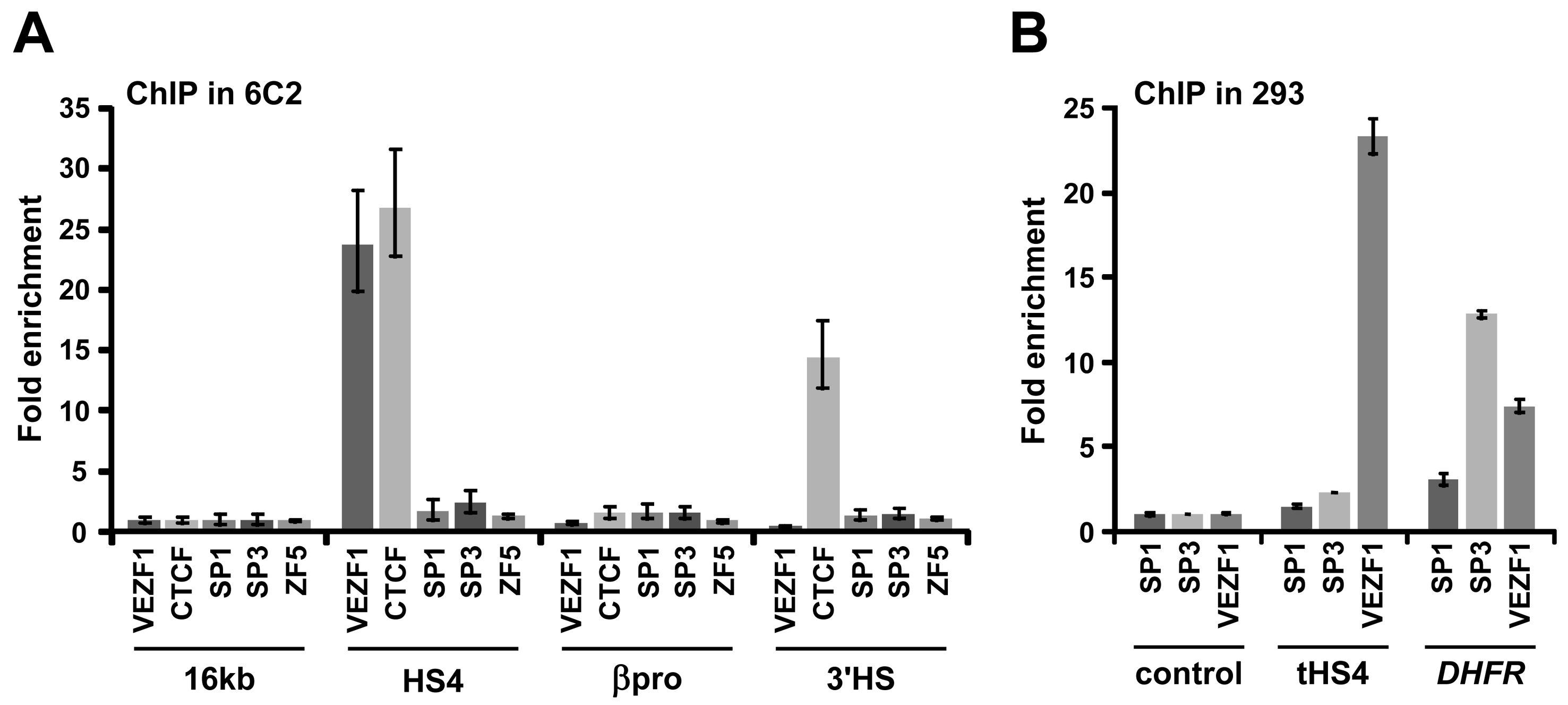

Supplement: Figure S5 — SP1, SP3, and ZF5 do not interact with HS4 in vivo. (A) ChIP analysis of transcription factor interactions at the β-globin locus in 6C2 erythroid progenitor cells. DNA enrichments at the {lower case beta}A promoter or the HS4 and 3′HS insulators were normalized to a negative control located in the 16 kb condensed chromatin region upstream of the β-globin locus. (B) ChIP analysis of transcription factor interactions with a stably integrated HS4 element in transgenic human 293 cells. Interactions with the DHFR CpG island promoter are shown as a positive control for SP1 and SP3 binding. DNA enrichments are normalized to an AT-rich negative control locus. (0.49 MB TIF) [file pgen.1000804.s005.tif]

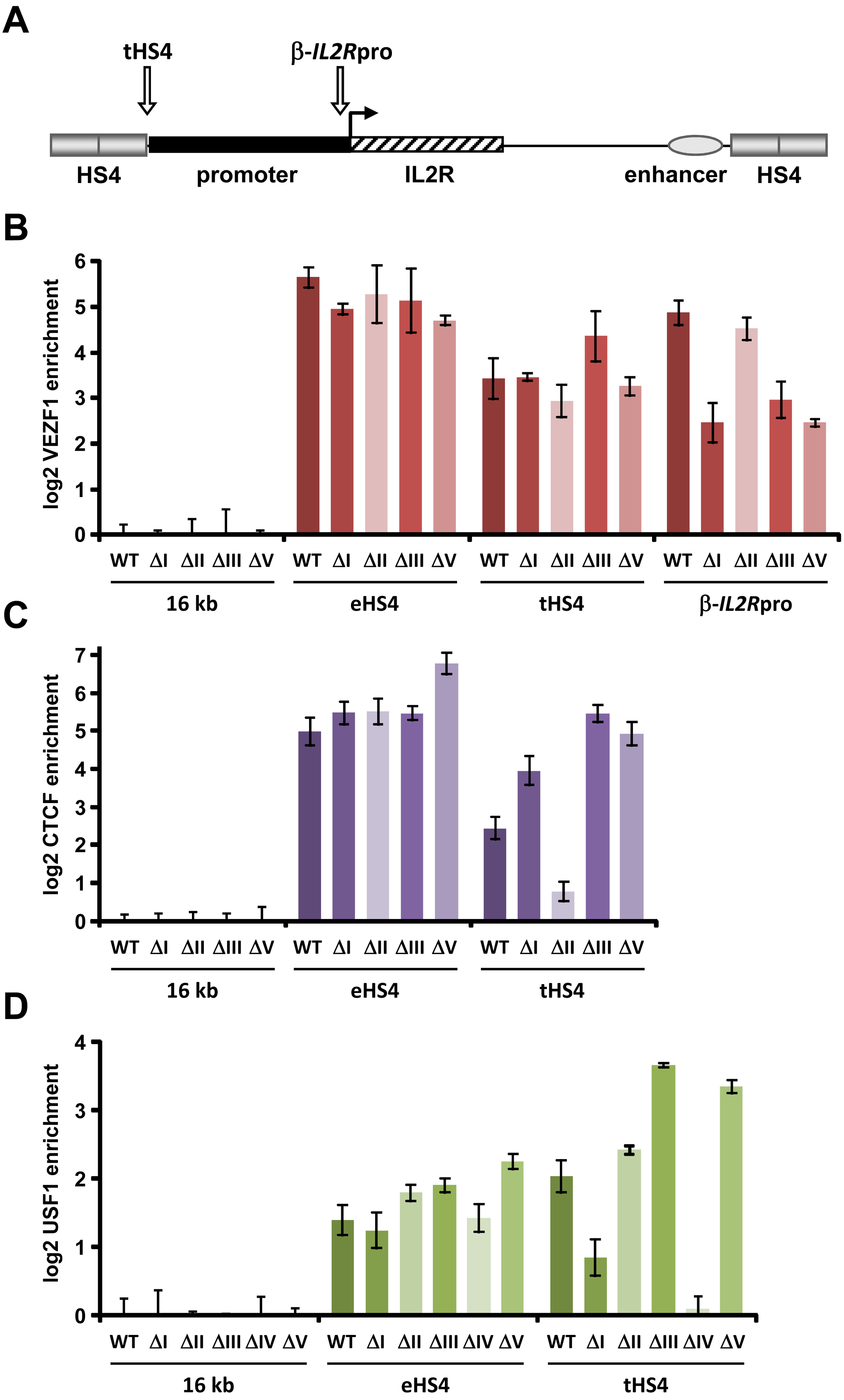

Supplement: Figure S6 — VEZF1 binding to HS4 is resistant to individual binding site mutations. (A) Schematic representation of the IL-2R transgene showing location of QPCR primer sets used to analyze the interaction of VEZF1 with the transgenic HS4 and promoter elements. (B–D) ChIP analysis of (B) VEZF1, (C) CTCF, or (D) USF1 interactions at the endogenous HS4 insulator (eHS4) and the transgenic HS4 (tHS4) and promoter (β-IL2Rpro) elements in the same 6C2 cell lines used for DNA methylation (Figure 3) and histone modification [14] analysis. DNA enrichments were normalized to a negative control located in the 16 kb condensed chromatin region upstream of the β-globin locus. Deletion of either footprint II or footprint IV disrupts CTCF and USF1 binding as expected. In contrast, deletion of individual VEZF1 sites footprint I, III, or V have no significant effect on overall VEZF1 ChIP efficiency. (1.09 MB TIF) [file pgen.1000804.s006.tif]

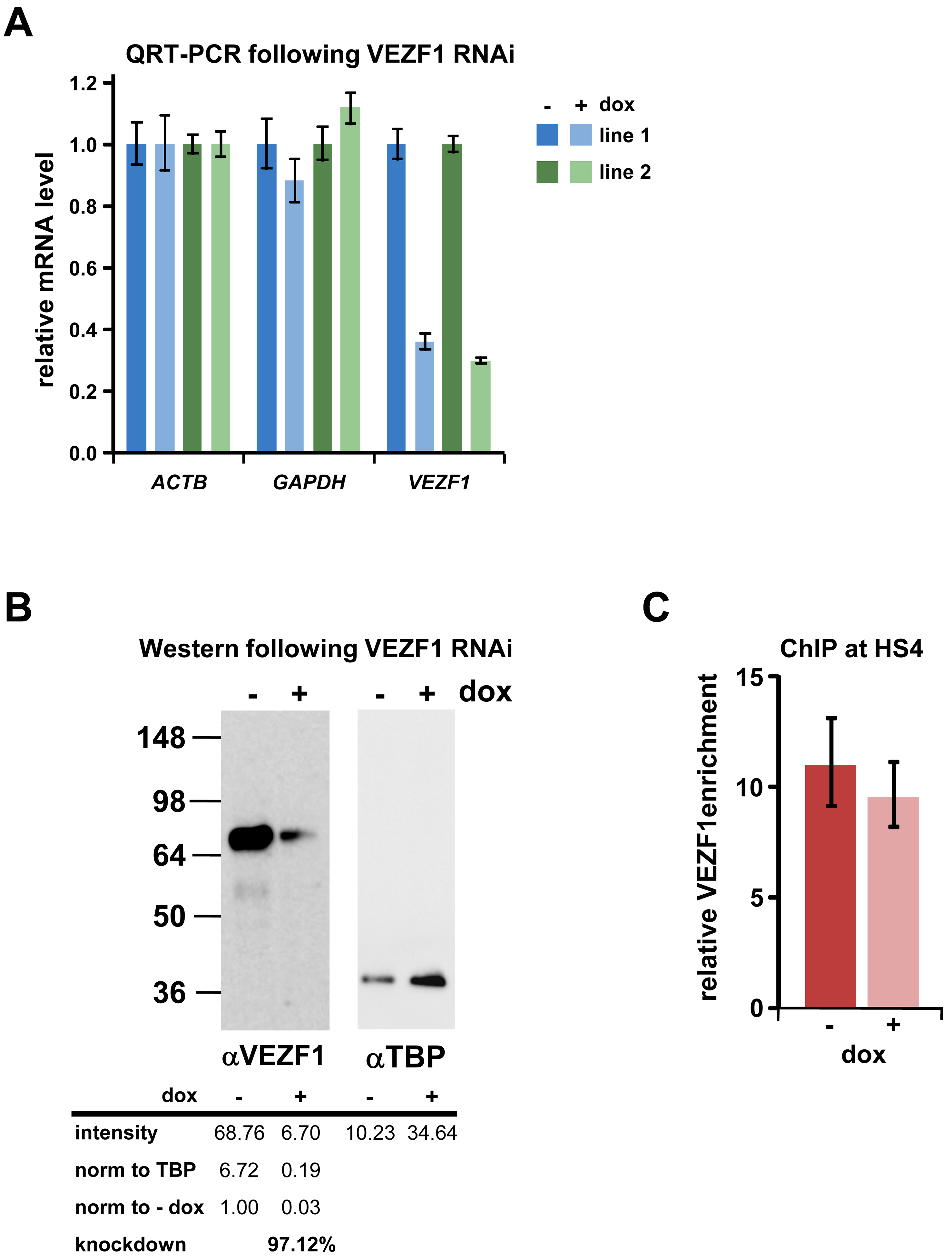

Supplement: Figure S7 — VEZF1 binding to HS4 is not significantly affected following VEZF1 RNAi. (A) Quantitative RT-PCR analysis following 48 hours of doxycycline-induction of two chicken 6C2 cell lines harboring lentiviral vectors that express VEZF1-specific miRNA. Expression levels are normalized to those of β-actin (ACTB) and untreated cells. VEZF1 mRNA levels are reproducibly knocked down by 70%. (B) Western blot analysis of chicken VEZF1 protein levels in one of the above lines with and without 14 days of doxycycline induction of VEZF1-specific miRNA. TBP levels were monitored as a loading control. VEZF1 and TBP band intensities visualized on a FUJI LAS3000 imager were quantified using AIDA software (shown below). Following normalization to TBP levels, VEZF1 protein levels were quantified to be knocked down by ∼97%. VEZF1-specific miRNA may cause translational inhibition in addition to the mRNA degradation observed by RT-PCR (C) ChIP analysis of VEZF1 interaction with the endogenous HS4 element following 14 days of induced VEZF1 knockdown. DNA enrichments were normalized to a negative control located in the 16 kb condensed chromatin region upstream of the β-globin locus. (1.64 MB TIF) [file pgen.1000804.s007.tif]

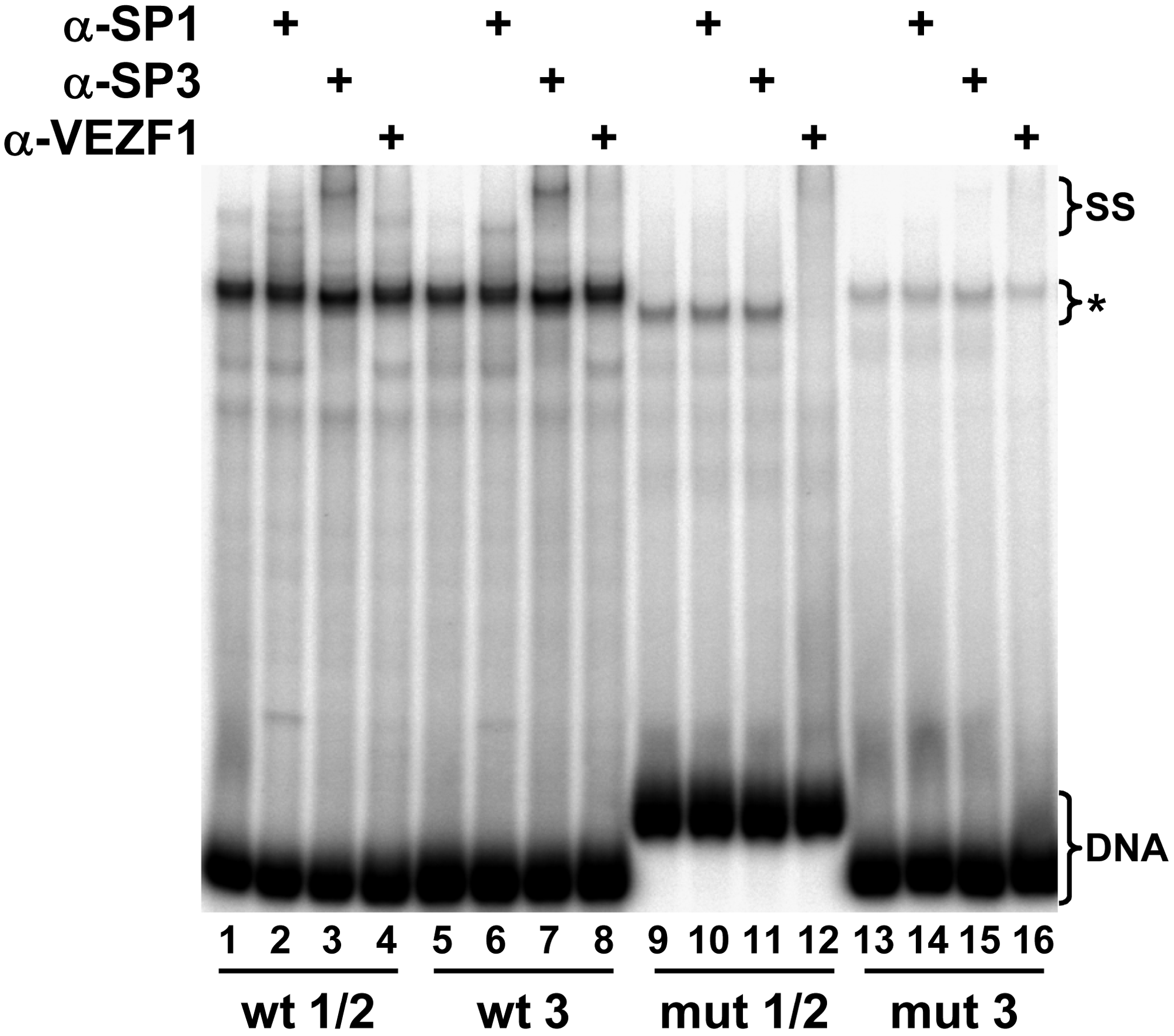

Supplement: Figure S8 — Transcription factor interactions with APRT CpG island elements. VEZF1 and SP1 interact with the wild type APRT CpG island, but the FIII mutant APRT element is only bound by VEZF1. Gel mobility supershift assays using 32P-labelled oligonucleotide duplexes containing either the wild type APRT SP1 sites 1&2 (lanes 1–4) and site 3 (lanes 5–8) or the mutant sites 1&2 (lanes 9–12) and site 3 (lanes 13–16). The core sequences of the duplexes are as shown in figure 8a. Nuclear extracts were pre-incubated with antibodies (indicated above each lane) prior to incubation with DNA. Supershifts are evidenced by the formation of low mobility ternary complexes (SS) in addition to abrogation of specific complexes (asterisk). Antibodies alone do not give rise to complexes with any of the duplexes used (not shown). SP1 and SP3 are detected in complexes with the wild type sites 1&2 and site 3 only. VEZF1 is detected in complexes with all four sites; weakest binding is seen at wild type sites 1&2 and strongest binding is seen at mutant sites 1&2. (1.78 MB TIF) [file pgen.1000804.s008.tif]
