# Supplementary material for: VEZF1 Elements Mediate Protection from DNA Methylation
Source: PLoS Genet. 2010 Jan 8;6(1):e1000804. doi: 10.1371/journal.pgen.1000804 (PMC2795164; doi:10.1371/journal.pgen.1000804)
Supplement: Table S1 — VEZF1 sites in the HS4 barrier protect a promoter from DNA methylation. CpG methylation of transgene promoters flanked by wild type or mutant HS4 insulators after 30 or 90 days of culture. The scoring of individual CpG bases from each clone subject to bisulfite sequencing is shown. Methylated bases are marked as ‘1’ and shaded blue. Average CpG methylation values are shown in Figure 5. Numbers above each table refer to CpG numbering from [16], where CpG 4–11 and 12–18 reside in the promoter and coding sequence, respectively. (0.02 MB PDF) [file pgen.1000804.s009.pdf]

Supplementary Table 1 (West)

Promoter at Day 30
Clones ordered by % methylation

6C2 line 8103 (WT HS4)

PROM	4	5	6	7	8	9	10	11	12	13	14	15	16	17	18	No. MeCpG
8103-2	0	0	0	0	0	0	0	0	0	0	1	0	0	0	0	1
8103-5	1	0	0	0	0	0	0	0	0	0	0	0	0	0	0	1
8103-1	0	0	0	0	0	0	0	0	0	0	0	0	0	0	0	0
8103-3	0	0	0	0	0	0	0	0	0	0	0	0	0	0	0	0
8103-4	0	0	0	0	0	0	0	0	0	0	0	0	0	0	0	0
8103-6	0	0	0	0	0	0	0	0	0	0	0	0	0	0	0	0
8103-7	0	0	0	0	0	0	0	0	0	0	0	0	0	0	0	0
8103-8	0	0	0	0	0	0	0	0	0	0	0	0	0	0	0	0
8103-9	0	0	0	0	0	0	0	0	0	0	0	0	0	0	0	0
8103-10	0	0	0	0	0	0	0	0	0	0	0	0	0	0	0	0
**% Meth**	**10**	**0**	**0**	**0**	**0**	**0**	**0**	**0**	**0**	**0**	**10**	**0**	**0**	**0**	**0**	**1.3%**

6C2 line 10401 (Δ1 HS4)

PROM	4	5	6	7	8	9	10	11	12	13	14	15	16	17	18	No. MeCpG
10401-9	1	1	1	1	1	1	1	1	1	1	1	1	1	1	1	15
10401-2	1	1	0	0	1	1	1	1	1	0	1	1	1	1	0	11
10401-4	1	1	1	1	1	1	0	0	0	0	1	1	1	1	0	10
10401-7	1	1	1	1	1	1	0	1	1	0	0	0	0	1	0	9
10401-10	0	1	1	1	1	1	1	1	0	0	0	0	0	0	1	8
10401-8	0	1	0	0	1	1	0	1	1	0	1	0	0	1	0	7
10401-5	0	0	0	1	1	1	0	0	1	0	0	0	1	1	0	6
10401-6	1	1	1	0	0	0	0	0	1	0	0	0	1	1	0	6
10401-1	0	0	0	0	1	0	0	0	0	0	0	0	0	0	0	1
10401-3	0	0	0	0	0	0	0	0	0	0	0	0	1	0	0	1
**% Meth**	**50**	**70**	**50**	**50**	**80**	**70**	**30**	**50**	**60**	**10**	**40**	**30**	**60**	**70**	**20**	**49.3%**

6C2 line 10506 (Δ2 HS4)

PROM	4	5	6	7	8	9	10	11	12	13	14	15	16	17	18	No. MeCpG
10506-6	0	0	0	0	0	0	0	0	0	0	1	0	0	0	0	1
10506-1	0	0	0	0	0	0	0	0	0	0	0	0	0	0	0	0
10506-2	0	0	0	0	0	0	0	0	0	0	0	0	0	0	0	0
10506-3	0	0	0	0	0	0	0	0	0	0	0	0	0	0	0	0
10506-4	0	0	0	0	0	0	0	0	0	0	0	0	0	0	0	0
10506-5	0	0	0	0	0	0	0	0	0	0	0	0	0	0	0	0
10506-7	0	0	0	0	0	0	0	0	0	0	0	0	0	0	0	0
10506-8	0	0	0	0	0	0	0	0	0	0	0	0	0	0	0	0
10506-9	0	0	0	0	0	0	0	0	0	0	0	0	0	0	0	0
10506-10	0	0	0	0	0	0	0	0	0	0	0	0	0	0	0	0
**% Meth**	**0**	**0**	**0**	**0**	**0**	**0**	**0**	**0**	**0**	**0**	**10**	**0**	**0**	**0**	**0**	**0.7%**

Supplementary Table 1 (West)

Promoter at Day 30

Clones ordered by % methylation

6C2 line 10615 (Δ3 HS4)

PROM	4	5	6	7	8	9	10	11	12	13	14	15	16	17	18	No. MeCpG
10615-1	1	1	1	1	1	1	1	1	1	1	1	1	1	1	1	15
10615-2	1	1	1	1	1	1	1	1	1	1	1	1	1	1	1	15
10615-4	1	1	1	1	1	1	1	1	1	1	1	1	1	1	1	15
10615-5	1	1	1	1	1	1	1	1	1	1	1	1	1	1	1	15
10615-6	1	1	1	1	1	1	1	1	1	1	1	1	1	1	1	15
10615-7	1	1	1	1	1	1	1	1	1	1	1	1	1	1	1	15
10615-8	1	1	1	1	1	1	1	1	1	1	1	1	1	1	1	15
10615-9	1	1	1	1	1	1	1	1	1	1	1	1	1	1	1	15
10615-10	1	1	1	1	1	1	1	1	1	1	1	1	1	1	1	15
10615-3	1	1	1	1	1	1	1	0	1	1	1	1	1	1	1	14
**% Meth**	**100**	**100**	**100**	**100**	**100**	**100**	**100**	**90**	**100**	**100**	**100**	**100**	**100**	**100**	**100**	**99.3%**

6C2 line 10901 (Δ4 HS4)

PROM	4	5	6	7	8	9	10	11	12	13	14	15	16	17	18	No. MeCpG
10901-3	0	1	0	0	1	0	0	0	0	0	0	0	1	1	0	4
10901-4	0	0	0	0	0	0	0	0	0	0	0	0	1	1	0	2
10901-1	0	0	0	0	0	0	0	0	0	0	0	0	0	0	0	0
10901-2	0	0	0	0	0	0	0	0	0	0	0	0	0	0	0	0
10901-5	0	0	0	0	0	0	0	0	0	0	0	0	0	0	0	0
10901-6	0	0	0	0	0	0	0	0	0	0	0	0	0	0	0	0
10901-7	0	0	0	0	0	0	0	0	0	0	0	0	0	0	0	0
10901-8	0	0	0	0	0	0	0	0	0	0	0	0	0	0	0	0
10901-9	0	0	0	0	0	0	0	0	0	0	0	0	0	0	0	0
10901-10	0	0	0	0	0	0	0	0	0	0	0	0	0	0	0	0
**% Meth**	**0**	**10**	**0**	**0**	**10**	**0**	**0**	**0**	**0**	**0**	**0**	**0**	**20**	**20**	**0**	**4.0%**

6C2 line 8d5 (Δ5 HS4)

PROM	4	5	6	7	8	9	10	11	12	13	14	15	16	17	18	No. MeCpG
8D5-1	1	1	1	1	1	1	1	1	1	1	1	1	1	1	0	14
8D5-2	1	1	1	1	1	1	1	1	1	1	1	1	1	1	0	14
8D5-4	0	1	1	1	1	1	0	1	1	1	1	1	1	1	1	13
8D5-3	1	1	1	1	1	1	0	0	0	0	0	0	0	0	0	6
8D5-9	1	0	0	0	1	1	0	1	0	0	0	0	1	1	0	6
8D5-6	0	0	0	0	0	0	1	0	1	0	1	1	1	0	0	5
8D5-7	1	1	1	0	1	1	0	0	0	0	0	0	0	0	0	5
8D5-10	1	1	1	1	0	1	0	0	0	0	0	0	0	0	0	5
8D5-5	0	1	1	0	0	0	0	0	0	0	0	0	0	0	0	2
8D5-8	0	0	0	0	0	0	0	0	0	0	0	0	0	1	0	1
**% Meth**	**60**	**70**	**70**	**50**	**60**	**70**	**30**	**40**	**40**	**30**	**40**	**40**	**50**	**50**	**10**	**47.3%**

Supplementary Table 1 (West)

Promoter at Day 90
Clones ordered by % methylation

6C2 line 8103 (WT HS4)

PROM	4	5	6	7	8	9	10	11	12	13	14	15	16	17	18	No. MeCpG
8103 - 3	0	0	0	0	0	1	0	0	0	0	1	0	0	0	0	2
8103 - 8	0	0	0	0	1	1	0	0	0	0	0	0	0	0	0	2
8103 - 5	0	0	0	0	0	0	0	0	0	0	0	0	0	0	1	1
8103 - 7	0	0	0	0	0	1	0	0	0	0	0	0	0	0	0	1
8103 - 9	0	0	0	1	0	0	0	0	0	0	0	0	0	0	0	1
8103 - 1	0	0	0	0	0	0	0	0	0	0	0	0	0	0	0	0
8103 - 2	0	0	0	0	0	0	0	0	0	0	0	0	0	0	0	0
8103 - 4	0	0	0	0	0	0	0	0	0	0	0	0	0	0	0	0
8103 - 6	0	0	0	0	0	0	0	0	0	0	0	0	0	0	0	0
8103 - 10	0	0	0	0	0	0	0	0	0	0	0	0	0	0	0	0
**% Meth**	**0**	**0**	**0**	**10**	**10**	**30**	**0**	**0**	**0**	**0**	**10**	**0**	**0**	**0**	**10**	**4.7%**

6C2 line 10401 (Δ1 HS4)

PROM	4	5	6	7	8	9	10	11	12	13	14	15	16	17	18	No. MeCpG
10401 - 2	1	1	1	1	1	1	1	1	1	1	1	1	1	1	1	15
10401 - 3	1	1	1	1	1	1	1	1	1	1	1	1	1	1	1	15
10401 - 1	1	1	1	1	1	1	1	1	1	1	1	1	1	1	0	14
10401 - 4	1	1	1	1	1	1	1	1	1	1	1	0	1	1	0	13
10401 - 7	1	1	1	1	1	1	0	1	1	1	1	1	1	1	0	13
10401 - 9	1	1	1	1	1	1	1	1	1	1	1	1	0	1	0	13
10401 - 8	1	1	1	1	1	1	0	1	1	1	0	1	1	1	0	12
10401 - 10	1	1	1	1	1	1	1	1	1	0	1	1	0	1	0	12
10401 - 5	1	1	0	1	1	1	1	1	1	0	1	1	1	0	0	11
10401 - 6	1	1	0	1	1	1	1	1	1	0	1	1	1	0	0	11
**% Meth**	**100**	**100**	**80**	**100**	**100**	**100**	**80**	**100**	**100**	**70**	**90**	**90**	**80**	**80**	**20**	**86.0%**

6C2 line 10506 (Δ2 HS4)

PROM	4	5	6	7	8	9	10	11	12	13	14	15	16	17	18	No. MeCpG
10506 1	0	0	0	0	0	0	0	0	0	0	0	0	0	0	1	1
10506 2	0	0	0	0	0	0	0	0	0	0	0	0	0	0	0	0
10506 3	0	0	0	0	0	0	0	0	0	0	0	0	0	0	0	0
10506 4	0	0	0	0	0	0	0	0	0	0	0	0	0	0	0	0
10506 5	0	0	0	0	0	0	0	0	0	0	0	0	0	0	0	0
10506 6	0	0	0	0	0	0	0	0	0	0	0	0	0	0	0	0
10506 7	0	0	0	0	0	0	0	0	0	0	0	0	0	0	0	0
10506 8	0	0	0	0	0	0	0	0	0	0	0	0	0	0	0	0
10506 9	0	0	0	0	0	0	0	0	0	0	0	0	0	0	0	0
10506 10	0	0	0	0	0	0	0	0	0	0	0	0	0	0	0	0
**% Meth**	**0**	**0**	**0**	**0**	**0**	**0**	**0**	**0**	**0**	**0**	**0**	**0**	**0**	**0**	**10**	**0.7%**

Supplementary Table 1 (West)

Promoter at Day 90
Clones ordered by % methylation

6C2 line 10615 (Δ3 HS4)

PROM	4	5	6	7	8	9	10	11	12	13	14	15	16	17	18	No. MeCpG
10615 - 4	1	1	1	1	1	1	1	1	1	1	1	1	1	1	1	15
10615 - 6	1	1	1	1	1	1	1	1	1	1	1	1	1	1	1	15
10615 - 10	1	1	1	1	1	1	1	1	1	1	1	1	1	1	1	15
10615 - 1	1	1	1	1	1	1	1	1	1	1	1	1	1	1	0	14
10615 - 2	1	0	1	1	1	1	1	1	1	1	1	1	1	1	1	14
10615 - 3	1	1	1	1	1	1	1	1	1	1	1	1	1	1	0	14
10615 - 5	1	1	1	1	1	1	0	1	1	1	1	1	1	1	1	14
10615 - 8	1	1	1	1	1	1	0	1	1	1	1	1	1	1	1	14
10615 - 9	1	1	1	1	1	1	1	1	1	0	1	1	1	1	0	13
10615 - 7	1	1	1	1	1	1	1	1	1	1	1	0	1	0	0	12
**% Meth**	**100**	**90**	**100**	**100**	**100**	**100**	**80**	**100**	**100**	**90**	**100**	**90**	**100**	**90**	**60**	**93.3%**

6C2 line 10901 (Δ4 HS4)

PROM	4	5	6	7	8	9	10	11	12	13	14	15	16	17	18	No. MeCpG
10901 - 3	1	0	1	1	1	1	0	0	1	1	0	0	0	0	0	7
10901 - 8	0	1	1	1	1	0	0	0	0	0	0	0	0	0	0	4
10901 - 4	0	0	0	0	0	0	0	0	1	1	0	0	0	0	0	2
10901 - 1	0	0	0	1	0	0	0	0	0	0	0	0	0	0	0	1
10901 - 6	1	0	0	0	0	0	0	0	0	0	0	0	0	0	0	1
10901 - 2	0	0	0	0	0	0	0	0	0	0	0	0	0	0	0	0
10901 - 5	0	0	0	0	0	0	0	0	0	0	0	0	0	0	0	0
10901 - 7	0	0	0	0	0	0	0	0	0	0	0	0	0	0	0	0
10901 - 9	0	0	0	0	0	0	0	0	0	0	0	0	0	0	0	0
10901 - 10	0	0	0	0	0	0	0	0	0	0	0	0	0	0	0	0
**% Meth**	**20**	**10**	**20**	**30**	**20**	**10**	**0**	**0**	**20**	**20**	**0**	**0**	**0**	**0**	**0**	**10.0%**

6C2 line 8d5 (Δ5 HS4)

PROM	4	5	6	7	8	9	10	11	12	13	14	15	16	17	18	No. MeCpG
8D5 - 1	1	1	1	1	1	1	1	1	1	1	1	1	1	1	1	15
8D5 - 10	1	1	1	1	1	1	1	1	1	1	1	1	1	1	1	15
8D5 - 2	1	1	1	1	1	1	1	1	1	1	1	0	1	1	1	14
8D5 - 4	1	1	1	1	1	1	1	1	1	0	1	1	1	1	1	14
8D5 - 5	1	1	1	1	1	1	0	1	1	1	1	1	1	1	1	14
8D5 - 8	0	1	1	1	1	1	1	1	1	1	1	1	1	1	1	14
8D5 - 9	0	1	1	1	1	1	1	1	1	1	1	1	1	1	1	14
8D5 - 3	1	1	1	1	1	1	0	0	1	0	1	1	1	1	1	12
8D5 - 6	1	0	1	1	1	1	0	1	1	0	1	1	1	1	0	11
8D5 - 7	1	0	1	1	1	1	0	1	1	0	1	1	1	1	0	11
**% Meth**	**80**	**80**	**100**	**100**	**100**	**100**	**60**	**90**	**100**	**60**	**100**	**90**	**100**	**100**	**80**	**89.3%**
