# Supplementary material for: VEZF1 Elements Mediate Protection from DNA Methylation
Source: PLoS Genet. 2010 Jan 8;6(1):e1000804. doi: 10.1371/journal.pgen.1000804 (PMC2795164; doi:10.1371/journal.pgen.1000804)
Supplement: Table S2 — Mutations of insulator protein binding sites result in the de novo methylation of HS4. CpG methylation of wild type (WT) or mutant (ΔI - ΔV) HS4 insulators after 30 or 90 days of culture. Both copies of HS4 were sequenced, except for ΔII and ΔV, where only the outermost copy was sequenced (see Materials and Methods). The scoring of individual CpG bases from each clone subject to bisulfite sequencing is shown. Methylated bases are marked as ‘1’ and shaded blue. Average CpG methylation values are shown in Figure 3. Numbers above each table refer to CpG numbering as assigned in Figure 3b. (0.03 MB PDF) [file pgen.1000804.s010.pdf]

Supplementary Table 2 (West)

Upstream HS4 insulators at Day 30

Clones ordered by % methylation

Outer copy of HS4

6C2 line 8103 (WT HS4)

	FI	FII								FIII								FIV				FV
**HS4**	**1a**	**2a**	**3a**	**4a**	**5a**	**6a**	**7a**	**8a**	**9a**	**10a**	**11a**	**12a**	**13a**	**14a**	**15a**	**16a**	**17a**	**18a**	**19a**	**20a**	**21a**	**22a**
8103-1	0	0	0	0	0	0	0	0	0	0	0	0	0	0	0	0	0	0	1	0	0	0
8103-2	0	0	0	0	0	0	0	0	0	0	0	0	0	0	0	0	0	0	0	1	0	0
8103-4	0	0	0	0	0	0	0	0	0	0	0	0	0	0	0	0	0	0	0	1	0	0
8103-7	0	0	0	0	0	0	0	0	0	0	0	0	0	0	0	0	0	0	0	0	0	0
8103-8	0	0	0	0	0	0	0	0	0	0	0	0	0	0	0	1	0	0	0	0	0	0
8103-3	0	0	0	0	0	0	0	0	0	0	0	0	0	0	0	0	0	0	0	0	0	0
8103-5	0	0	0	0	0	0	0	0	0	0	0	0	0	0	0	0	0	0	0	0	0	0
8103-6	0	0	0	0	0	0	0	0	0	0	0	0	0	0	0	0	0	0	0	0	0	0
8103-9	0	0	0	0	0	0	0	0	0	0	0	0	0	0	0	0	0	0	0	0	0	0
8103-10	1	0	0	0	0	0	0	0	0	0	0	0	0	0	0	0	0	0	0	0	0	0
**% Meth**	**10**	**0**	**0**	**0**	**0**	**0**	**0**	**0**	**0**	**0**	**0**	**0**	**0**	**0**	**0**	**10**	**0**	**0**	**10**	**20**	**0**	**0**

6C2 line 10401 (Δ1 HS4)

	FI	FII								FIII								FIV				FV
**HS4**	**1a**	**2a**	**3a**	**4a**	**5a**	**6a**	**7a**	**8a**	**9a**	**10a**	**11a**	**12a**	**13a**	**14a**	**15a**	**16a**	**17a**	**18a**	**19a**	**20a**	**21a**	**22a**
10401-8		0	0	0	0	0	0	0	0	0	0	0	1	0	0	0	1	0	0	0	0	0
10401-5		0	0	0	0	0	0	0	0	0	0	0	0	0	0	0	0	0	0	0	0	1
10401-10		0	0	0	0	0	0	0	0	0	0	0	0	0	0	0	0	0	0	0	0	0
10401-3		0	0	0	0	0	0	0	0	0	0	0	0	0	0	0	0	0	0	0	0	0
10401-4		0	0	0	0	0	0	0	0	0	0	0	0	0	0	0	0	0	0	0	0	0
10401-6		0	0	0	0	0	1	0	0	0	0	0	0	0	0	0	0	0	0	0	0	0
10401-1		0	0	0	0	0	0	0	0	0	0	0	0	0	0	0	0	0	0	0	0	0
10401-2		0	0	0	0	0	0	0	0	0	0	0	0	0	0	0	0	0	0	0	0	0
10401-7		0	0	0	0	0	0	0	0	0	0	0	0	0	0	0	0	0	0	0	0	0
10401-9		0	0	0	0	0	0	0	0	0	0	0	0	0	0	0	0	0	0	0	0	0
**% Meth**		**0**	**0**	**0**	**0**	**0**	**10**	**0**	**0**	**0**	**0**	**0**	**10**	**0**	**0**	**0**	**10**	**0**	**0**	**0**	**0**	**10**

6C2 line 10506 (Δ2 HS4)

	FI	FII								FIII								FIV				FV	
**HS4**	**1a**	**2a**	**3a**	**4a**	**5a**	**6a**	**7a**	**8a**	**9a**	**10a**	**11a**	**12a**	**13a**	**14a**	**15a**	**16a**	**17a**	**18a**	**19a**	**20a**	**21a**	**22a**	**Total**
10401 - 9	1			1	1	1	1	1	1	1	0	0	0	1	0	1	1	1	1	0	1	0	14
10401 - 8	1			1	1	1	0	1	1	1	1	1	0	1	1	1	0	1	0	0	0	1	14
10401 - 7	1			0	1	1	0	0	1	1	1	1	0	1	1	1	1	0	0	1	1	0	13
10401 - 3	1			0	1	1	1	0	1	1	1	0	0	0	1	1	0	1	1	1	0	1	13
10401 - 4	1			0	1	1	0	0	1	1	1	0	0	0	1	1	1	1	1	1	0	1	13
10401 - 5	0			0	1	0	0	0	1	1	1	0	0	0	1	1	1	1	1	1	0	1	11
10401 - 1	0			1	1	0	0	1	1	1	0	0	0	0	0	0	0	1	1	1	0	0	8
10401 - 10	1			0	0	0	0	0	0	0	1	0	0	1	1	0	1	0	1	0	1	0	7
10401 - 2	1			0	0	0	1	1	0	0	0	1	0	0	1	0	0	0	0	0	1	0	6
10401 - 6	0			0	0	1	0	0	0	0	0	0	0	0	0	0	0	0	0	0	0	0	1
**% Meth**	**70**			**30**	**70**	**60**	**30**	**40**	**70**	**70**	**60**	**30**	**0**	**40**	**70**	**60**	**50**	**60**	**60**	**50**	**40**	**40**	**50.00%**

6C2 line 10615 (Δ3 HS4)

	FV				FIV									FIII							FII	FI
**HS4**	**22a**	**21a**	**20a**	**19a**	**18a**	**17a**	**16a**	**15a**	**14a**	**13a**	**12a**	**11a**	**10a**	**9a**	**8a**	**7a**	**6a**	**5a**	**4a**	**3a**	**2a**	**1a**
10615-1	0	0	0	0	0	0	0	0	0	0	0			0	0	0	0	0	0	0	0	0
10615-4	0	0	0	0	0	0	0	0	0	0	0			0	0	0	0	0	0	0	0	0
10615-5	0	0	0	0	0	0	0	0	0	0	0			0	0	0	0	0	0	0	0	0
10615-6	0	0	0	0	0	0	0	0	0	0	0			0	0	0	0	0	0	0	0	0
10615-8	0	0	0	0	0	0	0	0	0	0	0			0	0	0	0	0	0	0	0	0
10615-10	0	0	0	0	0	0	0	0	0	0	0			0	0	0	0	0	0	0	0	0
10615-9	0	0	0	0	0	0	0	0	0	0	0			0	0	0	0	0	0	0	0	0
10615-3	0	0	0	0	0	0	0	0	0	0	0			0	0	0	0	0	0	0	0	0
10615-7	0	0	0	0	0	0	0	0	0	0	0			0	0	0	0	0	0	0	0	0
10615-2	0	0	0	0	0	0	0	0	0	0	0			0	0	0	0	0	0	0	0	0
**% Meth**	**0**	**0**	**0**	**0**	**0**	**0**	**0**	**0**	**0**	**0**	**0**			**0**	**0**	**0**	**0**	**0**	**0**	**0**	**0**	**0**

6C2 line 10901 (Δ4 HS4)

	FV				FIV									FIII							FII	FI
**HS4**	**22a**	**21a**	**20a**	**19a**	**18a**	**17a**	**16a**	**15a**	**14a**	**13a**	**12a**	**11a**	**10a**	**9a**	**8a**	**7a**	**6a**	**5a**	**4a**	**3a**	**2a**	**1a**
DC D4 7	0	0	0			1	1	1	1	1	0	1	1	1	1	1	1	1	0	0	0	0
DC D4 9	0	0	0			0	0	1	1	1	0	1	1	1	1	1	1	1	0	0	0	0
DC D4 4	0	0	0			0	0	1	0	0	1	1	1	1	1	1	1	1	0	0	0	0
DC D4 2	0	1	0			1	1	0	0	0	0	1	0	0	0	1	0	0	0	0	0	0
DC D4 1	0	0	1			0	0	0	1	0	0	0	0	1	1	1	0	0	0	0	0	0
DC D4 3	0	0	0			0	0	0	0	0	1	0	0	0	0	0	0	0	0	0	0	1
DC D4 5	0	0	0			0	0	1	1	1	0	0	0	0	1	0	0	0	0	0	0	0
DC D4 6	1	0	0			0	0	0	0	0	0	1	0	0	0	1	0	0	0	0	0	0
DC D4 8	0	0	0			0	0	0	0	0	0	0	0	1	1	0	0	0	0	0	0	0
DC D4 10	0	0	0			0	0	0	0	0	0	1	0	0	0	0	0	0	0	0	0	0
**% Meth**	**10**	**10**	**10**			**20**	**20**	**40**	**40**	**30**	**20**	**60**	**30**	**50**	**60**	**60**	**30**	**30**	**0**	**0**	**0**	**10**

6C2 line 8d5 (Δ5 HS4)

	FV				FIV									FIII							FII	FI	
**HS4**	**22a**	**21a**	**20a**	**19a**	**18a**	**17a**	**16a**	**15a**	**14a**	**13a**	**12a**	**11a**	**10a**	**9a**	**8a**	**7a**	**6a**	**5a**	**4a**	**3a**	**2a**	**1a**	**Total**
8D5-2		1	0	1	1	0	0	0	0	0	0	1	0	1	0	1	0	0	1	1	1	0	9
8D5-5		1	1	1	0	0	0	0	0	1	0	0	0	1	0	1	0	0	0	0	1	0	7
8D5-8		1	0	1	1	0	0	0	0	0	1	0	0	1	0	1	0	0	0	0	1	0	7
8D5-1		0	0	1	1	0	0	0	0	0	0	0	0	1	0	1	0	0	0	0	1	0	5
8D5-10		0	1	0	0	0	0	0	0	0	0	1	0	1	1	0	1	0	0	0	0	0	5
8D5-3		0	0	0	0	0	0	0	0	1	0	0	0	1	0	1	0	0	0	0	1	0	4
8D5-4		0	0	0	0	0	0	0	0	0	0	0	0	1	0	1	0	0	0	0	1	0	3
8D5-6		0	0	0	0	0	0	0	0	0	0	0	0	1	0	1	0	0	0	0	1	0	3
8D5-7		0	0	0	0	0	0	0	0	0	0	0	0	1	0	1	0	0	0	0	1	0	3
8D5-9		0	1	0	0	0	0	0	0	0	0	0	0	1	0	0	0	0	0	0	0	0	2
**% Meth**		**30**	**30**	**40**	**30**	**0**	**0**	**0**	**0**	**20**	**10**	**20**	**0**	**100**	**10**	**80**	**10**	**0**	**10**	**10**	**80**	**0**	**23%**

Supplementary Table 2 (West) **Upstream HS4 insulators at Day 30** **Inner copy of HS4**

Clones ordered by % methylation

6C2 line 8103 (WT HS4)

FI	FII							FIII									FIV				FV	
**1b**	**2b**	**3b**	**4b**	**5b**	**6b**	**7b**	**8b**	**9b**	**10b**	**11b**	**12b**	**13b**	**14b**	**15b**	**16b**	**17b**	**18b**	**19b**	**20b**	**21b**	**22b**	**Total**
0	0	0	0	0	0	0	0	0	0	0	0	0	0	0	0	0	0	0	0	0	1	2
1	0	0	0	0	0	0	0	0	0	0	0	0	0	0	0	0	0	0	0	0	0	1
1	0	0	0	0	0	0	0	0	0	0	0	0	0	0	0	0	0	0	0	0	0	1
0	0	0	0	0	0	0	0	0	1	0	0	0	0	0	0	0	0	0	0	0	0	1
0	0	0	0	0	0	0	0	0	0	0	0	0	0	0	0	0	0	0	0	0	0	1
0	0	0	0	0	0	0	0	0	0	0	0	0	0	0	0	0	0	0	0	0	0	0
0	0	0	0	0	0	0	0	0	0	0	0	0	0	0	0	0	0	0	0	0	0	0
0	0	0	0	0	0	0	0	0	0	0	0	0	0	0	0	0	0	0	0	0	0	0
0	0	0	0	0	0	0	0	0	0	0	0	0	0	0	0	0	0	0	0	0	0	0
0	0	0	0	0	0	0	0	0	0	0	0	0	0	0	0	0	0	0	0	0	0	0
**20**	**0**	**0**	**0**	**0**	**0**	**0**	**0**	**0**	**10**	**0**	**0**	**0**	**0**	**0**	**0**	**0**	**0**	**0**	**0**	**0**	**10**	**2%**

6C2 line 10401 (Δ1 HS4)

FI	FII							FIII									FIV				FV	
**1b**	**2b**	**3b**	**4b**	**5b**	**6b**	**7b**	**8b**	**9b**	**10b**	**11b**	**12b**	**13b**	**14b**	**15b**	**16b**	**17b**	**18b**	**19b**	**20b**	**21b**	**22b**	**Total**
	0	0	0	0	0	0	0	0	0	0	0	0	0	0	0	1	0	0	0	0	0	3
	0	0	0	0	0	0	0	0	0	0	0	0	0	0	0	1	0	0	0	0	0	2
	1	0	0	0	0	0	0	0	0	0	1	0	0	0	0	0	0	0	0	0	0	2
	0	0	0	0	0	0	0	0	0	0	0	0	0	0	0	0	1	0	0	0	0	1
	0	0	0	0	0	0	0	0	0	0	1	0	0	0	0	0	0	0	0	0	0	1
	0	0	0	0	0	0	0	0	0	0	0	0	0	0	0	0	0	0	0	0	0	1
	0	0	0	0	0	0	0	0	0	0	0	0	0	0	0	0	0	0	0	0	0	0
	0	0	0	0	0	0	0	0	0	0	0	0	0	0	0	0	0	0	0	0	0	0
	0	0	0	0	0	0	0	0	0	0	0	0	0	0	0	0	0	0	0	0	0	0
	0	0	0	0	0	0	0	0	0	0	0	0	0	0	0	0	0	0	0	0	0	0
	**10**	**0**	**0**	**0**	**0**	**0**	**0**	**0**	**0**	**0**	**20**	**0**	**0**	**0**	**0**	**20**	**10**	**0**	**0**	**0**	**0**	**2%**

6C2 line 10506 (Δ2 HS4)

SECOND CORE NOT ANALYZED

6C2 line 10615 (Δ3 HS4)

FV				FIV									FIII							FII	FI	
**22b**	**21b**	**20b**	**19b**	**18b**	**17b**	**16b**	**15b**	**14b**	**13b**	**12b**	**11b**	**10b**	**9b**	**8b**	**7b**	**6b**	**5b**	**4b**	**3b**	**2b**	**1b**	**Total**
0	0	0	0	0	0	0	0	0	0	0			0	0	0	1	0	0	0	0	0	1
0	0	0	0	0	0	0	0	0	0	0			0	0	0	0	1	0	0	0	0	1
0	0	0	0	0	0	0	0	0	0	0			0	0	0	1	0	0	0	0	0	1
0	0	0	0	0	0	0	0	0	0	0			0	0	0	1	0	0	0	0	0	1
0	0	0	0	0	0	0	0	0	0	0			1	0	0	0	0	0	0	0	0	1
0	0	0	0	0	0	0	0	0	0	0			0	0	0	0	0	0	0	0	1	1
0	0	0	0	0	0	0	0	0	0	0			0	0	0	1	0	0	0	0	0	1
0	0	0	0	0	0	0	0	0	0	0			0	0	0	0	0	0	0	0	0	0
0	0	0	0	0	0	0	0	0	0	0			0	0	0	0	0	0	0	0	0	0
0	0	0	0	0	0	0	0	0	0	0			0	0	0	0	0	0	0	0	0	0
**0**	**0**	**0**	**0**	**0**	**0**	**0**	**0**	**0**	**0**	**0**			**10**	**0**	**0**	**40**	**10**	**0**	**0**	**0**	**10**	**2%**

6C2 line 10901 (Δ4 HS4)

FV				FIV									FIII							FII	FI	
**22a**	**21a**	**20a**	**19a**	**18a**	**17a**	**16a**	**15a**	**14a**	**13a**	**12a**	**11a**	**10a**	**9a**	**8a**	**7a**	**6a**	**5a**	**4a**	**3a**	**2a**	**1a**	**Total**
0	0	0			0	0	1	1	1	1	0	0	0	0	0	0	0	0	0	0	0	16
0	0	0			0	0	0	0	0	1	1	0	0	0	0	0	0	0	0	0	0	12
0	0	0			0	0	0	0	0	1	1	0	0	0	0	0	0	0	0	0	0	11
0	0	0			0	0	0	0	0	1	0	0	0	0	1	0	0	0	0	0	0	7
0	0	0			0	0	0	0	0	0	0	0	0	0	0	0	0	0	0	0	0	5
0	0	0			0	0	1	1	0	0	0	0	0	0	0	0	0	0	0	0	0	4
0	0	0			0	0	0	0	0	0	0	0	0	0	0	0	0	0	0	0	0	4
0	0	0			0	0	0	0	0	1	0	0	0	0	1	0	0	0	0	0	0	4
0	0	0			0	0	0	0	0	0	0	0	0	0	1	0	0	0	0	0	0	3
0	0	0			0	0	0	0	0	0	0	0	0	0	0	0	0	0	0	0	0	1
**0**	**0**	**0**			**0**	**0**	**20**	**20**	**10**	**50**	**20**	**0**	**0**	**0**	**30**	**0**	**0**	**0**	**0**	**0**	**0**	**17%**

6C2 line 8d5 (Δ5 HS4)

SECOND CORE NOT ANALYZED

Supplementary Table 2 (West) **Upstream HS4 insulators at Day 90** **Outer copy of HS4**

Clones ordered by % methylation

6C2 line 8103 (WT HS4)

	FI	FII								FIII									FIV			FV
**HS4**	**1a**	**2a**	**3a**	**4a**	**5a**	**6a**	**7a**	**8a**	**9a**	**10a**	**11a**	**12a**	**13a**	**14a**	**15a**	**16a**	**17a**	**18a**	**19a**	**20a**	**21a**	**22a**
DC WT 3	0	0	0	0	0	0	0	0	0	1	0	0	0	0	0	0	0	0	0	0	0	0
DC WT 7	0	0	0	0	0	0	0	0	0	0	0	0	0	0	0	0	0	0	0	0	0	0
DC WT 8	0	0	0	0	0	0	0	0	0	1	0	0	0	0	0	0	0	0	0	0	0	0
DC WT 9	0	0	0	0	0	0	0	0	0	0	0	0	0	0	0	0	0	0	0	0	0	0
DC WT 10	0	0	0	0	0	0	0	0	0	0	0	0	0	0	0	0	0	0	0	0	0	0
DC WT 1	0	0	0	0	0	0	0	0	0	0	0	0	0	0	0	0	0	0	0	0	0	0
DC WT 2	0	0	0	0	0	0	0	0	0	0	0	0	0	0	0	0	0	0	0	0	0	0
DC WT 4	0	0	0	0	0	0	0	0	0	0	0	0	0	0	0	0	0	0	0	0	0	0
DC WT 5	0	0	0	0	0	0	0	0	0	0	0	0	0	0	0	0	0	0	0	0	0	0
DC WT 6	0	0	0	0	0	0	0	0	0	0	0	0	0	0	0	0	0	0	0	0	0	0
**% Meth**	**0**	**0**	**0**	**0**	**0**	**0**	**0**	**0**	**0**	**20**	**0**	**0**	**0**	**0**	**0**	**0**	**0**	**0**	**0**	**0**	**0**	**0**

6C2 line 10401 (Δ1 HS4)

	FI	FII								FIII									FIV			FV
**HS4**	**1a**	**2a**	**3a**	**4a**	**5a**	**6a**	**7a**	**8a**	**9a**	**10a**	**11a**	**12a**	**13a**	**14a**	**15a**	**16a**	**17a**	**18a**	**19a**	**20a**	**21a**	**22a**
10401 - 1		0	0	0	0	1	1	1	1	0	1	1	1	1	1	0	1	1	1	0	0	1
10401 - 2		0	0	0	0	0	0	0	0	1	0	0	0	0	0	0	0	0	1	0	0	1
10401 - 5		0	0	0	0	0	1	1	0	1	0	0	0	0	1	0	1	0	1	1	1	0
10401 - 7		0	0	0	0	0	0	0	0	0	0	0	0	0	0	0	0	0	1	1	1	0
10401 - 6		0	0	0	0	1	1	0	1	1	0	0	1	1	0	0	0	0	0	0	0	1
10401 - 9		1	0	0	0	0	0	0	0	1	1	1	0	1	1	0	0	0	0	0	0	0
10401 - 3		0	0	0	0	1	0	0	0	1	0	0	0	0	1	0	0	0	1	1	0	0
10401 - 4		0	0	0	0	0	0	0	0	0	0	0	0	0	0	0	0	0	0	0	1	1
10401 - 10		0	0	0	0	1	0	0	0	0	0	0	1	1	1	0	0	0	0	0	0	0
10401 - 8		0	0	0	0	0	0	0	0	0	0	0	0	0	0	0	0	0	0	0	1	0
**% Meth**		**10**	**0**	**0**	**0**	**40**	**30**	**20**	**20**	**50**	**20**	**20**	**30**	**40**	**50**	**0**	**20**	**10**	**50**	**30**	**40**	**40**

6C2 line 10506 (Δ2 HS4)

	FI	FII								FIII									FIV			FV	
**HS4**	**1a**	**2a**	**3a**	**4a**	**5a**	**6a**	**7a**	**8a**	**9a**	**10a**	**11a**	**12a**	**13a**	**14a**	**15a**	**16a**	**17a**	**18a**	**19a**	**20a**	**21a**	**22a**	**Total**
10401 - 2	1			0	1	1	1	1	1	1	1	1	0	1	1	1	1	1	1	1	1	1	18
10401 - 5	1			1	1	1	1	1	1	1	1	1	0	0	0	0	0	0	0	0	1	1	12
10401 - 1	1			0	0	1	1	1	1	1	0	0	0	1	0	0	1	1	1	1	1	0	12
10401 - 3	1			0	1	1	1	1	1	1	0	0	0	0	0	0	0	0	0	1	1	1	10
10401 - 7	1			1	0	1	1	1	1	1	0	0	0	0	0	0	0	0	0	1	1	1	10
10401 - 6	1			0	1	1	1	1	1	0	0	0	0	0	0	0	0	0	0	1	1	1	9
10401 - 8	1			0	1	1	1	0	1	1	0	0	0	0	0	0	0	0	0	1	1	1	9
10401 - 9	1			0	1	1	1	1	1	1	0	0	0	0	0	0	0	0	0	1	0	0	8
10401 - 10	1			0	0	1	1	1	1	0	0	0	0	0	0	0	0	0	0	1	1	0	7
10401 - 4	1			0	1	0	0	0	1	1	0	0	0	0	0	0	0	0	0	1	1	1	7
**% Meth**	**100**			**20**	**70**	**90**	**90**	**80**	**100**	**80**	**20**	**20**	**0**	**20**	**10**	**10**	**20**	**20**	**20**	**90**	**90**	**70**	**51.00%**

6C2 line 10615 (Δ3 HS4)

	FV				FIV									FIII							FII	FI
**HS4**	**22a**	**21a**	**20a**	**19a**	**18a**	**17a**	**16a**	**15a**	**14a**	**13a**	**12a**	**11a**	**10a**	**9a**	**8a**	**7a**	**6a**	**5a**	**4a**	**3a**	**2a**	**1a**
10615 - 6	0	0	0	0	0	0	0	0	0	0	0			0	1	0	0	0	0	0	0	0
10615 - 1	0	0	0	0	0	0	0	0	0	0	0			0	0	0	0	0	0	0	0	0
10615 - 2	0	0	0	0	0	0	0	0	0	0	0			0	0	0	0	0	0	0	0	0
10615 - 3	0	0	0	0	0	0	0	0	0	0	0			0	0	0	0	0	0	0	0	0
10615 - 4	0	0	0	0	0	0	1	0	0	0	0			0	0	0	0	0	0	0	0	0
10615 - 5	0	0	0	0	0	0	0	0	0	0	0			0	0	0	0	0	0	0	0	0
10615 - 7	0	0	0	0	0	0	0	0	0	0	0			0	0	0	0	0	0	0	0	0
10615 - 8	0	0	0	0	0	0	0	0	0	0	0			0	0	0	0	0	0	0	0	0
10615 - 9	0	0	0	0	0	0	0	0	0	0	0			0	0	0	0	0	0	0	0	0
10615 - 10	0	0	0	0	0	0	0	0	0	0	0			0	0	0	0	0	0	0	0	0
**% Meth**	**0**	**0**	**0**	**0**	**0**	**0**	**10**	**0**	**0**	**0**	**0**			**0**	**10**	**0**	**0**	**0**	**0**	**0**	**0**	**0**

6C2 line 10901 (Δ4 HS4)

	FV				FIV									FIII							FII	FI
**HS4**	**22a**	**21a**	**20a**	**19a**	**18a**	**17a**	**16a**	**15a**	**14a**	**13a**	**12a**	**11a**	**10a**	**9a**	**8a**	**7a**	**6a**	**5a**	**4a**	**3a**	**2a**	**1a**
10901 - 6	0	0	0			0	0	1	1	0	1	0	0	1	1	1	0	0	0	0	0	0
10901 - 8	0	0	0			0	1	1	0	1	1	0	0	1	1	1	0	0	0	0	0	1
10901 - 9	0	0	1			1	1	1	1	1	1	0	1	0	1	1	0	0	0	0	0	1
10901 - 2	0	0	0			1	1	1	1	1	0	1	1	1	1	0	0	0	0	0	0	0
10901 - 3	0	0	0			0	0	1	1	0	0	0	0	0	1	0	0	0	0	0	0	1
10901 - 1	0	0	1			1	1	0	0	0	0	0	0	0	0	0	0	0	0	0	0	0
10901 - 4	0	0	0			0	0	1	1	0	0	0	0	0	1	0	0	0	0	0	0	0
10901 - 5	0	0	1			0	1	1	1	0	0	0	0	1	0	0	0	0	0	0	0	0
10901 - 7	0	0	0			0	0	0	0	0	0	0	0	0	0	0	0	0	0	0	0	0
10901 - 10	0	0	0			0	0	0	0	0	0	0	0	0	0	0	0	0	0	0	0	0
**% Meth**	**0**	**0**	**30**			**30**	**50**	**70**	**60**	**30**	**30**	**10**	**20**	**40**	**60**	**30**	**0**	**0**	**0**	**0**	**0**	**30**

6C2 line 8d5 (Δ5 HS4)

	FV				FIV									FIII							FII	FI	Total
**HS4**	**22a**	**21a**	**20a**	**19a**	**18a**	**17a**	**16a**	**15a**	**14a**	**13a**	**12a**	**11a**	**10a**	**9a**	**8a**	**7a**	**6a**	**5a**	**4a**	**3a**	**2a**	**1a**	**13**
8D5 - 4		1	1	1	0	1	1	1	0	1	1	1	1	1	1	1	0	0	0	0	0	0	11
8D5 - 5		1	1	1	0	1	1	1	1	0	0	0	1	1	1	1	0	0	0	0	0	0	10
8D5 - 2		1	1	1	1	0	1	1	1	0	0	0	0	1	1	1	0	0	0	0	0	0	7
8D5 - 3		0	0	0	0	1	0	1	1	1	1	1	1	0	0	0	0	0	0	0	0	0	5
8D5 - 9		1	1	1	0	1	0	1	0	0	0	0	0	0	0	0	0	0	0	0	0	0	4
8D5 - 1		0	1	1	0	1	1	0	0	0	0	0	0	0	0	0	0	0	0	0	0	0	4
8D5 - 6		1	1	1	0	0	0	0	0	0	0	0	0	0	1	0	0	0	0	0	0	0	4
8D5 - 10		0	1	1	1	0	0	0	0	0	1	0	0	0	0	0	0	0	0	0	0	0	3
8D5 - 7		1	1	0	0	1	0	0	0	0	0	0	0	0	0	0	0	0	0	0	0	0	2
8D5 - 8		0	0	1	1	0	0	0	0	0	0	0	0	0	0	0	0	0	0	0	0	0	0.3
**% Meth**		**60**	**80**	**80**	**30**	**60**	**40**	**50**	**30**	**20**	**30**	**20**	**30**	**30**	**40**	**30**	**0**	**0**	**0**	**0**	**0**	**0**	**30.0%**

Supplementary Table 2 (West)

Upstream HS4 insulators at Day 90

Clones ordered by % methylation

Inner copy of HS4

6C2 line 8103 (WT HS4)

FI	FII							FIII									FIV				FV	
**1b**	**2b**	**3b**	**4b**	**5b**	**6b**	**7b**	**8b**	**9b**	**10b**	**11b**	**12b**	**13b**	**14b**	**15b**	**16b**	**17b**	**18b**	**19b**	**20b**	**21b**	**22b**	**Total**
0	0	0	0	0	0	0	0	0	0	0	0	0	0	0	0	0	0	0	0	0	0	1
0	0	0	0	0	0	0	0	0	0	0	0	0	0	0	0	0	1	0	0	0	0	1
0	0	0	0	0	0	0	0	0	0	0	0	0	0	0	0	0	0	0	0	0	0	1
0	0	0	0	0	0	0	0	0	0	0	0	0	0	0	0	1	0	0	0	0	0	1
0	0	0	0	0	0	0	0	0	0	0	0	0	0	0	1	0	0	0	0	0	0	1
0	0	0	0	0	0	0	0	0	0	0	0	0	0	0	0	0	0	0	0	0	0	0
0	0	0	0	0	0	0	0	0	0	0	0	0	0	0	0	0	0	0	0	0	0	0
0	0	0	0	0	0	0	0	0	0	0	0	0	0	0	0	0	0	0	0	0	0	0
0	0	0	0	0	0	0	0	0	0	0	0	0	0	0	0	0	0	0	0	0	0	0
0	0	0	0	0	0	0	0	0	0	0	0	0	0	0	0	0	0	0	0	0	0	0
**0**	**0**	**0**	**0**	**0**	**0**	**0**	**0**	**0**	**0**	**0**	**0**	**0**	**0**	**0**	**10**	**10**	**10**	**0**	**0**	**0**	**0**	**1%**

6C2 line 10401 (Δ1 HS4)

FI	FII							FIII									FIV				FV	
**1b**	**2b**	**3b**	**4b**	**5b**	**6b**	**7b**	**8b**	**9b**	**10b**	**11b**	**12b**	**13b**	**14b**	**15b**	**16b**	**17b**	**18b**	**19b**	**20b**	**21b**	**22b**	**Total**
	0	0	0	0	0	0	1	0	1	1	1	1	0	1	1	0	0	0	0	0	0	20
	0	0	0	1	1	1	1	0	0	0	1	0	0	1	1	1	1	1	0	1	0	14
	0	0	0	0	0	1	1	0	0	0	0	0	1	1	1	0	0	0	0	0	0	13
	0	0	0	0	0	1	1	0	1	1	1	1	0	1	0	0	0	1	0	0	0	11
	0	0	0	0	0	0	1	1	0	0	0	0	0	0	0	0	0	0	0	0	0	9
	0	0	0	1	0	0	1	1	0	0	0	0	0	0	0	0	0	0	0	0	0	9
	0	0	0	0	0	1	0	0	0	0	0	0	0	0	0	0	0	0	0	0	0	6
	0	0	0	0	0	1	1	0	0	0	1	0	0	1	0	0	0	0	0	0	0	6
	0	0	0	0	0	0	0	0	0	0	0	0	0	0	0	0	0	1	0	0	0	5
	0	0	0	0	0	0	0	0	0	0	1	0	0	0	0	0	0	0	0	0	0	2
	**0**	**0**	**0**	**20**	**10**	**50**	**70**	**20**	**20**	**20**	**50**	**20**	**10**	**50**	**30**	**10**	**10**	**30**	**0**	**10**	**0**	**23%**

6C2 line 10506 (Δ2 HS4)

SECOND CORE NOT ANALYZED

6C2 line 10615 (Δ3 HS4)

FV				FIV									FIII							FII	FI	
**22b**	**21b**	**20b**	**19b**	**18b**	**17b**	**16b**	**15b**	**14b**	**13b**	**12b**	**11b**	**10b**	**9b**	**8b**	**7b**	**6b**	**5b**	**4b**	**3b**	**2b**	**1b**	**Total**
0	0	0	0	0	0	0	0	0	0	0			0	0	0	0	0	0	0	0	0	1
0	0	0	0	0	0	0	0	0	0	0			0	0	0	0	0	0	0	0	0	0
0	0	0	0	0	0	0	0	0	0	0			0	0	0	0	0	0	0	0	0	0
0	0	0	0	0	0	0	0	0	0	0			0	0	0	0	0	0	0	0	0	0
0	0	0	0	0	0	0	0	0	0	0			0	0	0	0	0	0	0	0	0	0
0	0	0	0	0	0	0	0	0	0	0			0	0	0	0	0	0	0	0	0	0
0	0	0	0	0	0	0	0	0	0	0			0	0	0	0	0	0	0	0	0	0
0	0	0	0	0	0	0	0	0	0	0			0	0	0	0	0	0	0	0	0	0
0	0	0	0	0	0	0	0	0	0	0			0	0	0	0	0	0	0	0	0	0
0	0	0	0	0	0	0	0	0	0	0			0	0	0	0	0	0	0	0	0	0
**0**	**0**	**0**	**0**	**0**	**0**	**0**	**0**	**0**	**0**	**0**			**0**	**0**	**0**	**0**	**0**	**0**	**0**	**0**	**0**	**1%**

6C2 line 10901 (Δ4 HS4)

FV				FIV									FIII							FII	FI	
**22b**	**21b**	**20b**	**19b**	**18b**	**17b**	**16b**	**15b**	**14b**	**13b**	**12b**	**11b**	**10b**	**9b**	**8b**	**7b**	**6b**	**5b**	**4b**	**3b**	**2b**	**1b**	**Total**
0	1	0			0	1	0	0	0	0	0	1	0	0	1	0	0	0	0	0	0	5
0	0	0			0	0	0	0	0	0	0	1	0	0	0	0	0	0	0	0	0	5
0	1	1			0	0	1	0	0	0	0	0	0	1	0	0	0	0	0	0	0	5
0	1	1			1	0	1	1	1	1	0	0	0	0	0	0	0	0	0	0	0	3
0	0	0			0	0	1	1	0	0	0	0	0	1	0	0	0	0	0	0	0	3
0	0	0			0	0	0	0	0	0	0	0	0	1	1	0	0	0	0	0	0	2
0	0	0			0	0	1	0	0	0	0	0	0	1	0	0	0	0	0	0	0	2
0	0	0			0	0	1	0	0	0	0	1	0	0	0	0	0	0	0	0	0	2
0	0	0			0	0	0	0	0	1	0	0	0	0	0	0	0	0	0	0	0	0
0	0	0			0	0	0	0	0	0	0	0	0	0	0	0	0	0	0	0	0	0
**0**	**30**	**20**			**10**	**10**	**50**	**20**	**10**	**20**	**0**	**30**	**0**	**40**	**20**	**0**	**0**	**0**	**0**	**0**	**0**	**19%**

6C2 line 8d5 (Δ5 HS4)

SECOND CORE NOT ANALYZED
