# Supplementary material for: VEZF1 Elements Mediate Protection from DNA Methylation
Source: PLoS Genet. 2010 Jan 8;6(1):e1000804. doi: 10.1371/journal.pgen.1000804 (PMC2795164; doi:10.1371/journal.pgen.1000804)
Supplement: Text S1 — DNA binding activities of candidate HS4-binding proteins; RNA interference methods; western blotting methods. (0.04 MB DOC) [file pgen.1000804.s011.doc]

**Supplementary text**

**DNA binding activities of candidate HS4-binding proteins**

We studied other candidate HS4-binding proteins isolated by DNA affinity purification. Supershift analysis shows that in addition to VEZF1, SP1 and SP3 are also components of complexes formed between nuclear extracts and the FI or the β^A^ sites *in vitro* (Figure S3A and D). Recombinant chicken SP1 and SP3 interact with the FI and β^A^ sites, with some specificity towards the (dG)_9_ string of FI (Figure S4). The central sequence of FI (CCCCCCCCC) is similar to the canonical SP1 binding site (CCCGCCC). However, SP1 motifs are poor competitors for FI complexes formed with nuclear extracts (Figure 4A, compare lanes 1 and 7). Recombinant SP1 or SP3 do not interact with footprints FIII or FV (Figure S4, data not shown). Nuclear protein complexes formed with FIII or FV are not supershifted with SP1 or SP3 antibodies, and are not competed by SP1 consensus duplexes (Figure S3, data not shown).

We conclude that SP1 and SP3 factors can interact with the contiguous dG-dC strings in the FI and β^A^ promoter VEZF1 sites *in vitro*, but do not recognize the bipartite VEZF1 sites in FIII or FV. In contrast to the *in vitro* gel mobility shift data, ChIP assays did not detect significant SP1 or SP3 interaction with HS4 in chicken cells (Figure S5A). We also assayed for SP1 and SP3 binding to a transgenic HS4 element (tHS4) that is stably integrated into human HEK293 cells. Minimal enrichments for SP1 and SP3 were observed at tHS4 that were not significantly greater than a negative control locus and were far smaller than observed at the *DHFR* promoter (Figure S5B). Conversely, enrichments for VEZF1 at HS4 are very high in all experiments. Thus, while VEZF1, SP1 and SP3 compete for binding to the FI site *in vitro,* it appears that VEZF1 is the predominant binding factor *in vivo*.

The zinc finger protein ZF5 was also isolated by both FI and FIII DNA affinity purification. However, we find that recombinant ZF5 only interacts with the FIII site *in vitro* and does not show any specificity towards the dG-dC string or any footprinted bases (Figure S4). Antibodies against ZF5 do not supershift complexes between nuclear extracts and the FI, FIII or FV sites (data not shown). Furthermore, we did not detect any binding of ZF5 to the HS4 element in ChIP assays (Figure S5A). We did not detect HSP70 or TEF1α in any of the complexes with FI, FIII or FV by supershift analysis (data not shown).

**RNA interference methods.**

Chicken 6C2 erythroleukaemia cells were grown in αMEM supplemented with 10 % FCS, 2 % chicken serum, 1 mM HEPES, 25 μM β-mercaptoethanol and 1 % Penicillin/Streptomycin solution. siRNAs were designed against chicken VEZF1 using the RNAi central webtool (http://katahdin.cshl.org:9331/RNAi_web/scripts/main2.pl).

Following guidelines (Mittal V (2004) Nat Rev Genet 5: 355-365), we focused on siRNAs that had GC at the 5’, AT at the 3’ and a T at position 10 and selected four siRNAs that had no near matches in the chicken genome (www.ensembl.org). siRNAis (Qiagen) were transfected into 6C2 cells (Lipofectamine 2000, Invitrogen) and chicken VEZF1 expression analyzed by RT-PCR and Western blotting. GgVEZF11242 was the best performing siRNA (Qiagen), with consistent knockdown in the region of 90%.

GgVEZF1-4_sense 5’GCUGCUAACUUGUGCCAAAdTdT
GgVEZF1-4_anti 5’UUUGGCACAAGUUAGCAGCdTdT

In order to assess the effects of long term knockdown of VEZF1 on barrier activity and DNA methylation patterns we assessed inducible systems for stable interfering RNA production. We found that systems which express short hairpin RNAs from RNA polymerase III promoters did not perform well in chicken 6C2 cells, primarily because cells did not maintain stable RNAi (not shown). We have had most success with a miR30-based lentiviral system (Shin K.J. et al. (2006) Proc Natl Acad Sci U S A 103: 13759-13764). The GgVEZF1242 trigger sequence was cloned into pSLIK-Venus. pSLIK-Venus was co-transfected with pMDLg/pRRE, pRSV-Rev and pCMV-VSV-G into 293T cells to produce viral particles. Lentivirally transduced 6C2 cells were cloned following flow sorting or serial dilution. GFP-miRNA expression was induced with 2 μg/ml doxycycline. GFP expression confirmed expression of the miRNA cassette. RT-PCR analysis indicated that chicken VEZF1 was reproducibly knocked down in the 6C2 line VEZF1-4/C3 by ~70%, whereas Western blotting indicated a knockdown of 97%.

**Western Blotting methods.**

Membranes were blocked in PBS, 0.05% Tween20, 5% non-fat dried milk and washed three times with PBS, 0.05% Tween20. Anti-VEZF1 (3642) antibodies were used at 1/2000 dilution in PBS, 0.05% Tween20, 5% non-fat dried milk overnight at 4^o^C. HRP conjugated goat anti-rabbit secondary antibody was used at 1/100000 dilution in PBS, 0.05% Tween20, 5% non-fat dried milk for 2 hours at room temperature. Blots were visualized by enhanced chemiluminescence (Pierce) detected on an FLA3000 (Fuji) and quantified using AIDA software.
